# Mitoxantrone alters CD24/Siglec-10 expression in malignant brain tumor models

**DOI:** 10.1038/s41598-026-37588-7

**Published:** 2026-01-28

**Authors:** Jan Kopecky, Julio Enríquez Pérez, Stevanus Jonathan, Tom Milos, Poi Kwanyuen, Julia Biskupiak, Edward Visse, Kelin Gonçalves De Oliveira, Hugo Talbot, Myriam Cerezo-Magaña, Valeria Governa, Jinquan Cai, Marie Arsenian Henriksson, Nils Ståhl, David Cederberg, Mattias Belting, Peter Siesjö, Anna Darabi

**Affiliations:** 1 Department of Clinical Sciences Lund, Neurosurgery, Lund University, Lund, Sweden; 2 Department of Clinical Sciences Lund, Oncology, Lund University, Lund, Sweden; 3 Department of Microbiology, Tumor and Cell Biology, Biomedicum B7, Karolinska Institutet, Stockholm, Sweden; 4 Department of Laboratory Medicine, Translational Cancer Research, Lund University, Lund, Sweden; 5 Department of Immunology, Genetics, and Pathology, Science for Life Laboratory, Uppsala University, Uppsala, Sweden; 6 Department of Hematology, Oncology, and Radiation physics, Skåne University Hospital, Lund, Sweden; 7 Department of Neurosurgery, Skåne University Hospital, Lund, Sweden; 8 Glioma Immunotherapy Group, Barngatan 4, Lund, 221-85 Sweden

**Keywords:** Cancer, Immunology, Biomarkers

## Abstract

Medulloblastoma and glioblastoma are the most common malignant primary brain tumors in children and adults, respectively. Tumor-associated macrophages and microglia are key non-cancerous cell types in these tumors. These cells interact with CD24, a so called “don’t eat me signal” expressed on tumor cells, through Siglec-10, a receptor that contributes to immune evasion by promoting an immunosuppressive environment. The CD24/Siglec-10 interaction in context of malignant brain tumors has been scarcely studied.In silico analyses reveal that *CD24* gene expression correlates with specific gene signatures associated with prognosis in both medulloblastoma and glioblastoma. In both human- and mouse brain tumors, Siglec-10^+^ cells co-express the microglia-associated molecule TREM2. Treatment with mitoxantrone as an immunogenic cell-death-inducing cytostatic agent led to a dose-dependent reduction in cell viability and cell surface CD24 levels in both murine and human brain tumor cell cultures. Intratumoral mitoxantrone administration in a murine CD24-high glioma model extended survival, decreased tumor size, reduced Siglec-10^+^/TREM2^+^ cell populations, and increased anti-tumor CD8^+^ cells. These findings suggest that targeting the CD24/Siglec-10 axis with mitoxantrone may modulate the tumor microenvironment and enhance anti-tumor immunity. **Keywords**: CD24, Siglec-10, Mitoxantrone, Malignant brain tumor, Immunotherapy.

**Supplementary Information:**

The online version contains supplementary material available at 10.1038/s41598-026-37588-7.

Abbreviations:

MB: medulloblastoma.

GBM: glioblastoma.

CNS: central nervous system.

C: classical.

M: mesenchymal.

PN: proneural.

IDHmut: isocitrate dehydrogenase mutant.

WHO: World Health Organization.

SHH: sonic hedgehog.

TAMs: tumor-associated macrophages and microglia.

ICI: immune checkpoint inhibitors.

MTX: mitoxantrone.

ICD: immunogenic cell death.

ATP: adenosine triphosphate.

HMGB1: high mobility group box-1.

BMDM: bone-marrow-derived macrophages.

MG: microglia.

hMB: human MB.

hGBM: human GBM.

## Introduction

Medulloblastoma (MB) is the most common malignant brain tumor in young children, with a 5-year (5y) survival rate of about 70%, though infants face an even poorer prognosis^1^. Glioblastoma (GBM) is the most prevalent malignant brain tumor in adults, with a dismal 5y survival rate of 3–6%^1,2^. In children, GBM is rare and has a 5y survival rate of less than 20%^3^. Current standard treatments, including maximal surgical resection, radiation, and chemotherapy, lead to severe side effects in children and only marginally extend survival in adults^2,4^. Brain tumor entities can be further divided into molecular subgroups that predict prognosis and represent distinct disease entities, underscoring the need for a more subgroup specific analysis and therapy to improve survival in brain tumor patients. In 2010 Verhaak et al., divided GBMs into the classical (C), mesenchymal (M) and proneural (PN) subgroup^5^. The PN subgroup also contained the isocitrate dehydrogenase (IDH)-mutant tumors, however these tumors have now been reclassified and are according to the latest world health organization (WHO) classification from 2021, called Astrocytoma, IDH-mutant^6^. Second-generation molecular subgrouping of MBs suggest a division into WNT, sonic hedgehog (SHH), and Non-WNT/Non-SHH Group 3 (Gr3) and Group 4 (Gr4) tumors, where the more heterogenous Gr3/Gr4 can be further divided into subtypes based on previous suggested classification of MBs^7–10^.

Given the extensive resistance of both MB and GBM to immune responses, it is crucial to explore the full spectrum of immune suppression mechanisms in these tumors to develop effective therapies. Immune profiling indicates that brain tumors are less immunologically active compared to other cancers^11–13^. Whether this is due to tumor-induced immune suppression, such as increased anti-phagocytic signals, or the fact that brain tumors are immunologically cold remains unclear. Nevertheless, both GBM and MB show subgroup-specific immune-profiles with a notable presence of immune-suppressive tumor-associated macrophages and microglia (TAMs)^14–16^. SHH MBs have a distinct macrophage expression profile, making them particularly promising targets for TAM-focused therapies.

Immune checkpoint inhibitors (ICI) that block the immune suppression related to adaptive immune cells are used to treat both solid tumors and hematological malignancies. However, these treatments are not effective for all cancer types and their success depends on the cell composition and function of the tumor immune-microenvironment. Tumors often evade immune detection through enhanced expression of anti-phagocytic signals, such as CD24 on tumor cells and Siglec-10 on TAMs. This interaction inhibits macrophage-mediated phagocytosis and Toll-like receptor-mediated inflammation in tumors, among other complex immune escape mechanisms^17,18^. Targeting of these interactions has recently gained attention, with inhibitors of the CD24-Siglec-10 axis emerging as potential new immunotherapies^18–20^.

CD24 is a heavily glycosylated protein, with its various glycosylation patterns being tissue-specific and believed to mediate its diverse functions^21^. During brain development, CD24 is expressed on differentiating neurons, and in the adult brain it is associated with cell proliferation during secondary neurogenesis^22^. In various cancers, CD24 are linked to worse prognosis, stemness, expression on tumor microvesicles, increased migration of tumor cells and tumor cell proliferation^23–25^. Mitoxantrone (MTX) is an anti-neoplastic agent with less cardiotoxicity than its analogue doxorubicin. MTX interfere with topoisomerase II resulting in inhibition of cell proliferations due to double stranded DNA-breaks^26^. MTX is also known to induce immunogenic cell death (ICD)^27^, resulting in the release of adenosine triphosphate (ATP), surface expression of calreticulin, and nuclear translocation of high mobility group box 1 (HMGB1). However, it is not routinely used in malignant brain tumor chemotherapy due to its poor lipid solubility and limited CNS penetration, though local administration directly into the tumor or the surgical cavity has been explored in several studies^28–31^.

Subgroup specific gene-expression patterns of *CD24* and *SIGLEC-10* in brain tumors have previously been presented^32–34^. However, due to the complex nature of brain tumors and the limited availability of models, especially in the field of pediatric brain tumors, research aimed at understanding the targeting of CD24 and Siglec-10 in representative brain tumor models is highly warranted. Based on our previous data from 813 brain tumor tissues and 178 normal CNS tissues, we observed that *CD24* gene expression was significantly upregulated in various brain tumors, with the highest levels in SHH, Group3, and Group4 MB. CD24 expression was maintained in cultured cells and in a MB Group3-xenograft model^33^. The goal of this study is to investigate the potential of targeting the CD24/Siglec-10 axis in the treatment of malignant brain tumors using relevant in vitro and in vivo models.

## Results

### *CD24* is associated with genes predicting prognosis in brain tumors

Understanding CD24-tumor heterogeneity is crucial for effective biomarker targeting. We first investigated the association of CD24 with a 10-gene high-risk signature known to predict poor prognosis (*FOXM1*,* NEK2*,* CCT2*,* ACTL6A*,* CCND2*,* ABL1*,* SYNCRIP*,* ITGB*,* ENAH*,* UMPS*) and a 10-gene low-risk signature known to predict a better prognosis (*ADAM22*,* AEBP1*,* DHRS2*,* RAC3*,* SHANK1*,* CYB5D2*,* IL27RA*,* DNAH2*,* ZPF3*,* NRXN2*) in MB, GBM, and other tumors^35–43^. We hypothesized that an increased positive correlation with genes predicting poor prognosis, and an increased negative correlation with genes predicting a better prognosis should indicate an elevated expression of CD24 by tumor cells. To test this hypothesis, we also included markers we believed should identify tumor cells, as the proliferation marker *MKI67* and for MB the tumor subgroup-specific genes *SMO* for SHH, *MYC* for Group3 and *CDK6* for Group 4^44^. To conduct these analyses, we utilized multiple publicly available datasets, as detailed in the Material and Methods section.

**Fig. 1:**
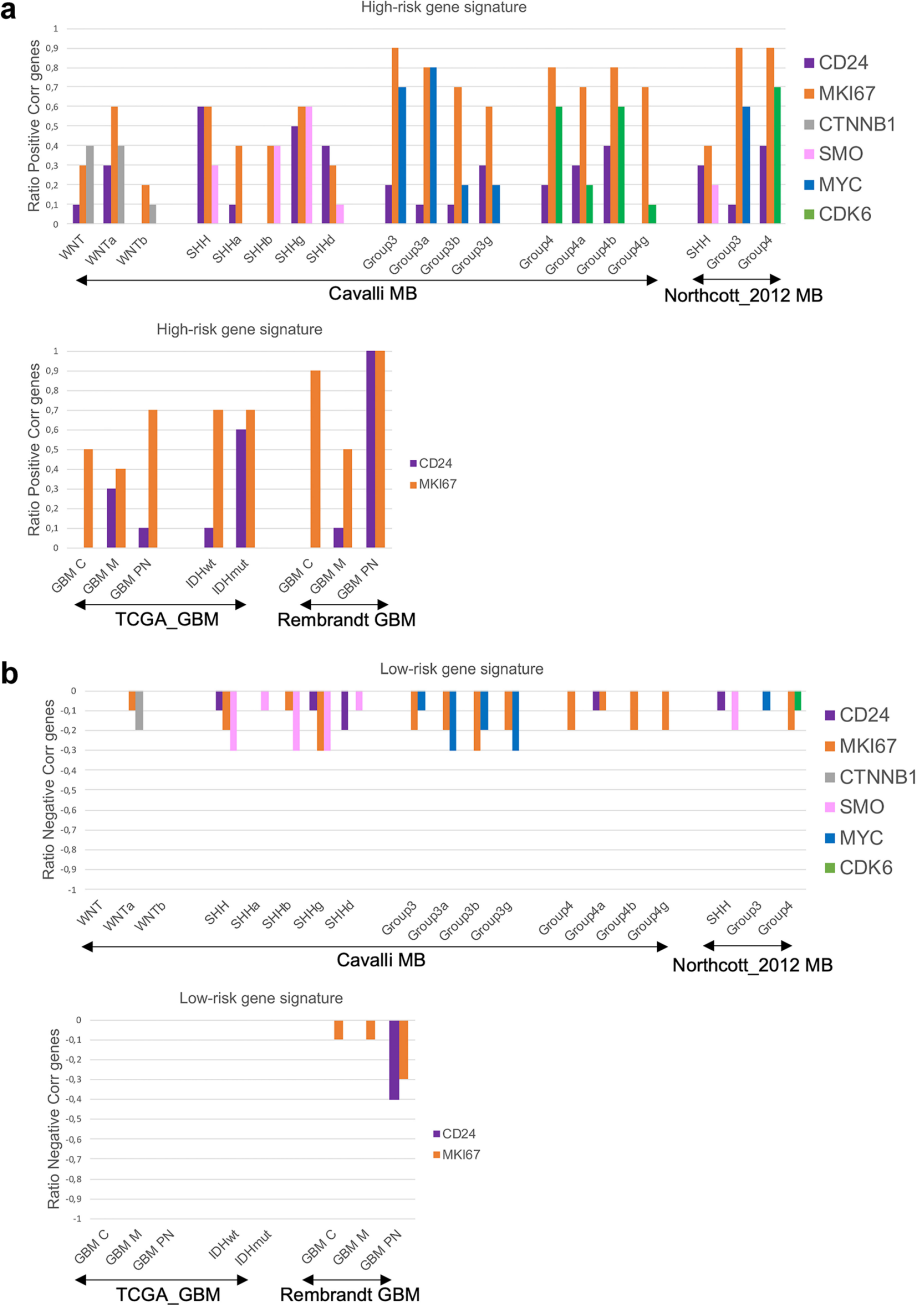
Association between *CD24* and genes predicting prognosis in brain tumors.

*CD24* had the strongest positive correlation with the 10-gene high-risk signature in SHH and Group4 MB, with Cavalli-SHHγ and Group4ß showing the highest correlations, as well as in IDHmut and GBM^PN^. As expected, *MKI67* showed strong positive correlations with most of the 10 selected high-risk genes, with subgroup-specific variations. For MB, subgroup specific genes also correlated to a high degree with the selected 10-gene high-risk signature (Fig. 1a). A negative correlation was detected between the 10-gene low-risk signature and *MKI67* in MB and in GBM (Rembrandt), and with the MB-subgroup specific genes *SMO* and *MYC*. *CD24* was negatively correlated with this signature only in SHH and in GBM^PN^ (Rembrandt) (Fig. 1b). For detailed Spearman correlation data, see Supplementary Table S1. Single-cell RNA sequencing confirmed that *CD24* is primarily expressed in the tumor cell compartment of medulloblastoma (SHH, Group 3, and Group 4) and gliomas. To a lesser extent, *CD24* was also detected in various immune cell types within these tumors. MB-data were obtained from the publicly available *Pediatric Neuro-oncology Cell Atlas* browser, and glioma data were visualized using the *UCSC Cell Browser* (see Supplementary Fig. S1).

In summary, our analysis indicates that *CD24* is associated with genes previously validated as poor prognostic markers in brain tumors, with differences depending on tumor type and subgroup. *CD24* is predominantly expressed by tumor cells, but to a lesser extent also by immune cells—a factor that should be considered when specifically targeting CD24 in malignant brain tumors.

For each subgroup, a Spearman correlation test was performed between *CD24* and the 10-gene signatures representing **(a)** high-risk; *FOXM1*,* NEK2*,* CCT2*,* ACTL6A*,* CCND2*,* ABL1*,* SYNCRIP*,* ITGB*,* ENAH*,* UMPS*, or **(b)** low-risk; *ADAM22*,* AEBP1*,* DHRS2*,* RAC3*,* SHANK1*, CYB5D2*,* IL27RA*,* DNAH2*, ZPF3*,* NRXN2*. The number of correlated genes for *CD24* was divided with 10 and the ratio was plotted in a histogram. The same was performed for *MKI67* (MB, GBM, IDHwt, IDHmut) and for genes representing different MB subgroups (WNT*-CTNNB1*, SHH-*SMO*, Group3-*MYC*, Group4-*CDK6*). A Spearman r-value of 0.26–0.29 (*p*-value < 0.001), and 0.30 or above (*p*-value < 0.05) was considered a positive correlation. A Spearman r-value of −0.26-(-)0.29 (*p*-value < 0.001), and − 0.30 or below (*p*-value < 0.05) was considered a negative correlation. Multiple publicly available datasets, as detailed in the Material and Methods section was used. Genes marked with* were not available in the TCGA_GBM dataset and the ratio was calculated by division with 8.

### SIGLEC-10 gene expression is correlated with microglia markers in human MB and GBM and display subgroup specific TAM-phenotypes

**Fig. 2:**
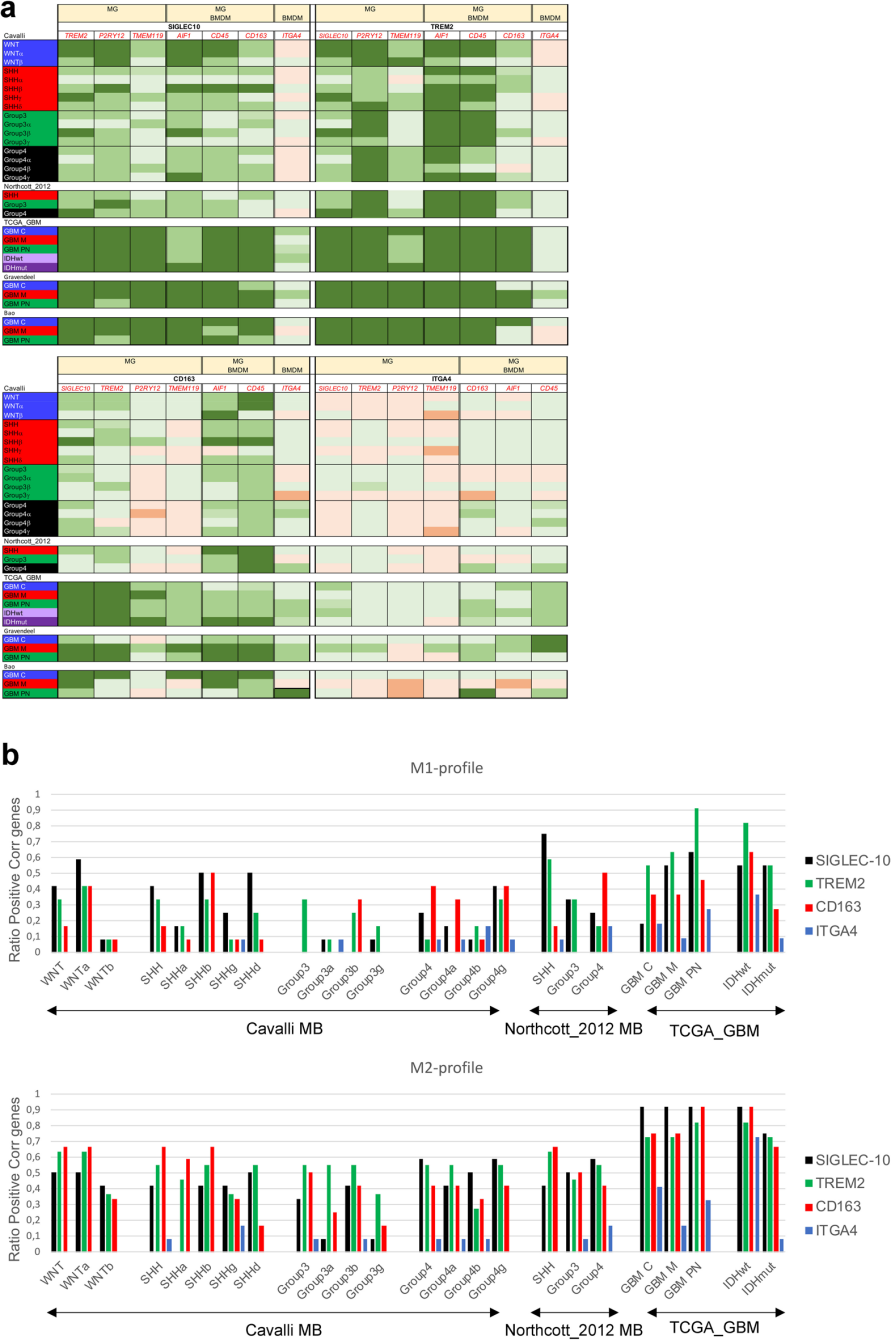
Spearman correlation test between Siglec-10 and genes representing MG and TAM-phenotypes in brain tumors.

We next wanted to elucidate subgroup-specific differences in *SIGLEC-10* expression, and the association with genes for tissue resident microglia (MG) through *TREM2*,* P2RY12*,* TMEM119* and *AIF1*, or bone-marrow-derived macrophages (BMDM) via *ITGA4*. CD163, *AIF1* (IBA1) and *PTPRC* (CD45) are expressed by both MG and BMDM. We found that *SIGLEC-10* correlated with *TREM2*,* P2RY12*,* AIF1*,* CD163*, and *PTPRC* (CD45) in both MB and GBM. The MG-marker *TMEM119* was strongly correlated with *SIGLEC-10* in GBM, but not in MB, and the correlation with the BMDM marker *ITGA4* was weak and only present in certain GBM subtypes. *TREM2* showed a strong correlation with the MG-specific marker *P2RY12* and with the shared MG/BMDM-markers *AIF1* and *PTPRC*, but less with *TMEM119*. Additionally, *CD163* and *ITGA4* had a weaker association with MG markers in MB compared to in GBM (Fig. 2a). Analysis of single-cell RNA sequencing data from MB and glioma confirmed a similar pattern. In addition to being expressed by BMDM, *ITGA4* was also associated with other immune cell types, such as T cells (Supplementary Fig. S1), which could explain its lower association with MG and TAMs.

We next investigated the expression of *SIGLEC-10*, *TREM2* (MG), *CD163* (shared between MG and BMDM), and *ITGA4* (BMDM marker) in relation to different TAM phenotypes using the CIBERSORT software^45^ as detailed in materials and Methods. We selected 12 key genes for each cell type to represent anti-tumoral M1, and pro-tumoral M2 TAMs. Overall, our analysis revealed that *SIGLEC-10*, *TREM2*, and *CD163* were more strongly associated with the M2 TAM profile than with M1 profiles. Notably, in SHH tumors *SIGLEC-10* showed a higher association with the M1 profile compared to M2. When examining M1 and M2 profiles separately, *SIGLEC-10* and *TREM2* were more associated with anti-tumoral M1 markers than *CD163* across several subgroups, including WNT, SHH, Group3, GBM^M^, GBM^PN^, and IDHmut. In contrast, *SIGLEC-10* and *TREM2* were more associated with the M2 profile in Group4 MB and all GBM subtypes, while *CD163* showed a stronger M1 profile in Group4 MB compared to *SIGLEC-10* and *TREM2* (Fig. 2b). Overall, these findings suggest that *SIGLEC-10* is a marker for MG in both MB and GBM and exhibits tumor- and subgroup-specific TAM phenotypes. For detailed Spearman correlation data, see Supplementary Table S1.

**2a**. MG (*TREM2*,* P2RY12*,* TMEM119*,* AIF1*), MG/BMDM (*AIF1*,* CD163*,* CD45*) and BMDM (*ITGA4*). A Spearman r-value equal to or above 0.26 (light green) and a *p*-value < 0.001 was considered a weak positive correlation, Spearman r equal to 0.30 or above (green) and a *p*-value < 0.05 a moderate positive correlation and Spearman r equal to 0.50 (dark green) or above and a *p*-value < 0.05 a strong positive correlation. A Spearman r equal to or below − 0.26 (light orange) and a *p*-value < 0.001 was considered a weak negative correlation, Spearman r equal to −0.30 or below (orange) and a *p*-value < 0.05 a moderate negative correlation and Spearman r equal to −0.50 (dark orange) or below and a *p*-value < 0.05 a strong negative correlation.

**2b.** Spearman correlation test between *SIGLEC-10*,* TREM2*,* CD163*, or *ITGA4* gene-expression and the 12 selected genes representing M1 and M2 cells was performed. For specific genes, see material and methods. The ratio of positively correlated genes per gene of interest (as defined above) to the total number of genes representing each cell profile was calculated and plotted. Multiple publicly available datasets, as detailed in the Material and Methods section was used.

### CD24^+^ and Siglec-10^+^ cells interact in human MB and GBM tissue

**Fig. 3:**
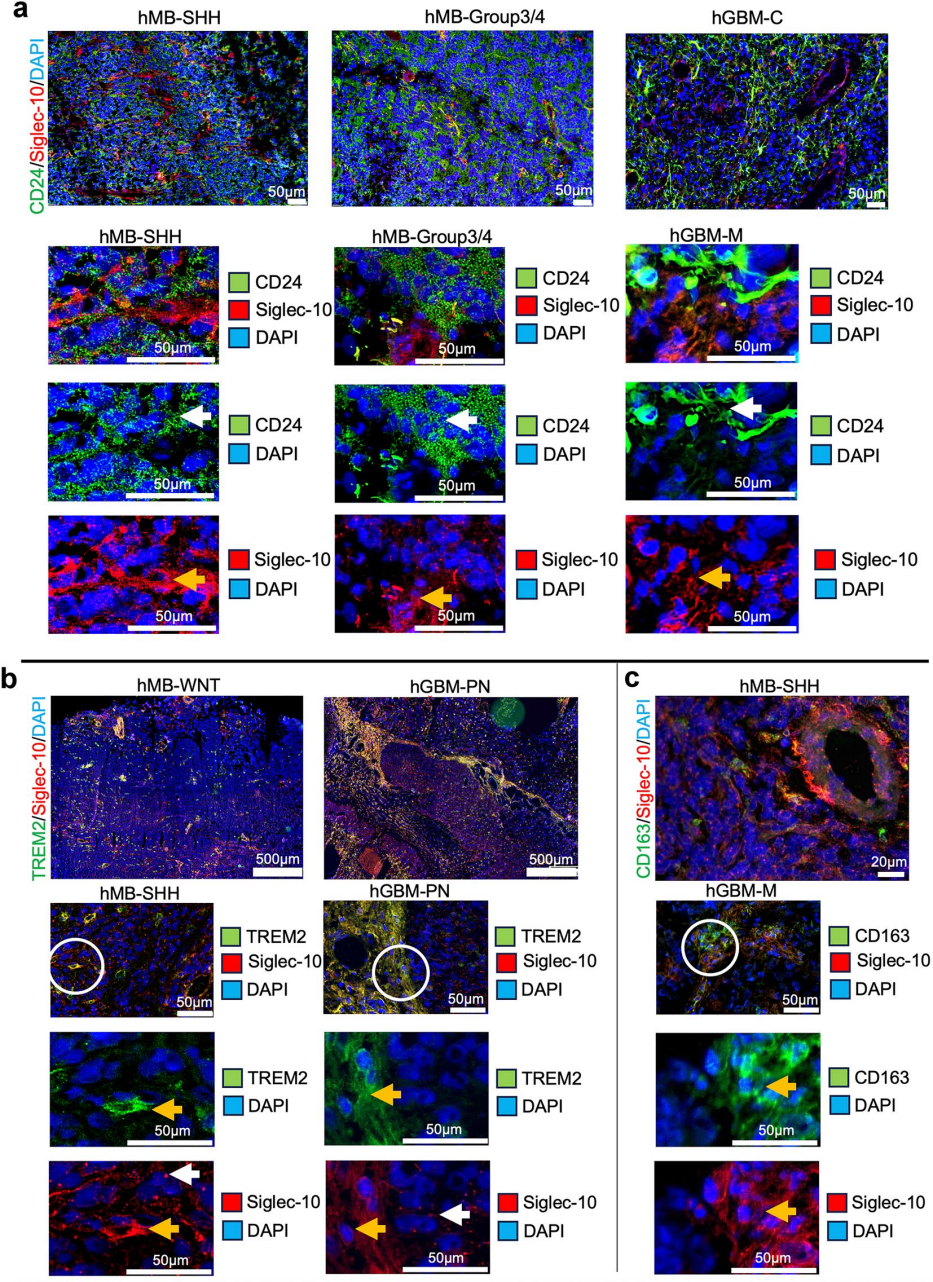
CD24^+^ and Siglec-10^+^ cells interact in human MB and GBM tissue.

To investigate CD24 and Siglec-10 interactions in vivo, human MB (hMB) and human GBM (hGBM) tissue were analyzed. Representative images from selected hMB- and hGBM subgroups are presented in Fig. 3. In hMB tissues, CD24 staining was uniformly distributed throughout the tissue, appearing in larger or smaller vesicle-like structures. Conversely, hGBM tissues exhibited a patchy CD24 expression pattern, with CD24^+^ cells displaying long protrusions. These observations align with our previously published data on tumor tissue and cultured hMB and hGBM cells^33^. Siglec-10^+^ cells interacted with cells expressing CD24 in both hMB and in hGBM (Fig. 3a, white arrows indicate staining on tumor cells, yellow arrows indicate staining on MG/TAMs). Dot plot analysis of gene expression levels of *CD24*,* SIGLEC-10*,* TREM2*,* P2RY12*,* TMEM119*,* AIF1*,* PTPRC* (CD45), *CD163*,* ITGA4* (CD49d) across MB and GBM subgroups confirmed the interaction between CD24 and Siglec-10, patterns suggest that CD24⁺ tumor cells and immune cell populations (e.g., TAMs and MG) are present within the same tumor microenvironment (Supplementary Fig. S2). This supports the plausibility of interactions between CD24⁺ tumor cells and these immune cell types, which may have implications for therapeutic strategies targeting CD24. To confirm that Siglec-10 represent MG/TAMs in situ, we also analyzed the co-localization of Siglec-10 with TREM2 and CD163. In hMB and hGBM, Siglec-10^+^ cells with long protrusions colocalized with TREM2 and in most cases with CD163. In hMB as well as in hGBM, no co-labeling was seen between Siglec-10 (long protrusions/pointy pattern) and the marker for BMDM - ITGA4 (CD49d). However, a few double-stained Siglec-10^+^/CD49d^+^ cells with TAM-morphology were found near blood vessels (Supplementary Fig. S3).

In summary this histological data confirms the gene-expression data stating that Siglec-10^+^ cells detected in brain tumors express MG/TAM-markers but not markers for BMDM. Tumor cells expressing CD24 and Siglec-10^+^TREM2^+^ cells interact both in hMB and in hGBM.

Selected tissue sections were stained using immunohistochemistry.

**a.** Upper panel: Representative spatial overviews of hMB (SHH, Group3/4) (10x magnification) and hGBM (M) (20x magnification). White circles indicate areas shown as enlarged picture below. CD24(green)/Siglec-10(red). DAPI was used as nuclear staining. Scale bar, 500 μm.

Lower panel: Enlarged pictures of hMB (SHH, Group3/4) and hGBM (M). White arrows indicate labeling on tumor cells. Yellow arrows indicate labeling on MG/TAMs. CD24(green)/Siglec-10(red). DAPI was used as nuclear staining. Scale bar, 50 μm.

**b.** Upper panel: Representative spatial overviews (10x magnification) of hMB (WNT) and hGBM (PN). TREM2(green)/Siglec-10(red). DAPI was used as nuclear staining. Scale bar, 500 μm.

Lower panel: Representative pictures (20x magnification) of hMB (SHH) and hGBM (PN). White circles indicate areas shown as enlarged picture below. White arrows indicate labeling on tumor cells. Yellow arrows indicate labeling on MG/TAMs. TREM2(green)/Siglec-10(red). DAPI was used as nuclear staining. Scale bar, 50 μm.

**c.** Upper panel: Representative spatial overview (10x magnification) of hMB (SHH). CD163(green)/Siglec-10(red). DAPI was used as nuclear staining. Scale bar, 500 μm.

Lower panel: Representative pictures (20x magnification) of hGBM (M). White circle indicates areas shown as enlarged picture below. Yellow arrows indicate labeling on TAMs.

### Intra-tumoral delivery of MTX prolongs survival and reduces tumor size in SB28^CD24high^ gliomas

**Fig. 4:**
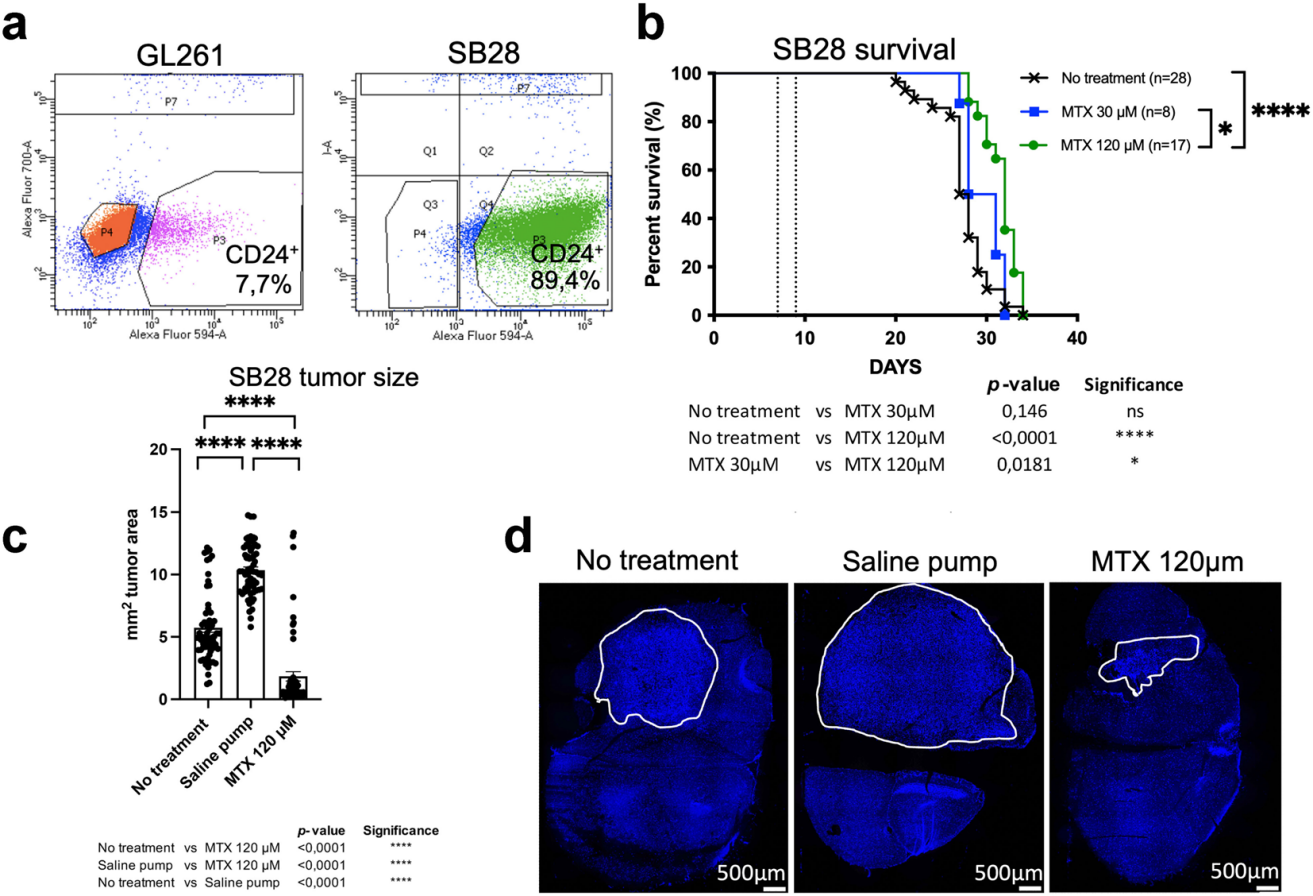
Analysis of CD24 expression, survival and tumor size in mouse gliomas4a.

We evaluated CD24 expression in two mouse glioma models: the immunogenic GL261 and the treatment-resistant SB28 cell lines. Flow-cytometric analysis revealed that GL261 cells expressed low levels of CD24 (7.7%), whereas SB28 cells exhibited high levels of CD24 (89.4%) (Fig. 4a). CD24 staining was confirmed on cultured cells, which showed very weak CD24 expression in GL261 cells compared to strong, vesicular CD24 labeling in SB28 cells, mirroring the CD24 expression pattern observed in human medulloblastoma (hMB). When cultured under stem-cell conditions in serum-free medium and on non-adherent flasks for 48–96 h, SB28 cells formed spheroids, whereas GL261 cells did not (Supplementary Fig. S4). We wanted to investigate MTX-targeting in brain tumors expressing increased levels of CD24. Therefore, the SB28^CD24high^ mouse glioma model was chosen.

MTX was administered directly into the tumor of SB28 bearing mice to improve drug delivery and to minimize systemic side effects, including dampening of the immune response. To evaluate the therapeutic effects of continuous intra-tumoral delivery of MTX, mice with established intracranial SB28^CD24high^ gliomas were treated using mini-osmotic pumps delivering MTX at concentrations of 30 µM (SB28, *n* = 8), or 120 µM (SB28, *n* = 17). Survival rates were compared with untreated controls (SB28, *n* = 28). All untreated mice developed lethal tumors. Though some treatment efficacy was observed, none of the MTX-treated mice survived. Statistically significant differences in survival were found between untreated controls and 120 µM MTX (no treatment *versus* 120 µM MTX, *p* < 0.0001), as well as between 30 µM and 120 µM MTX treatments (30 µM MTX *versus* 120 µM MTX, *p* = 0.0181). No significant difference was observed between untreated controls and 30 µM MTX (no treatment *versus* 30 µM MTX, *p* = 0.146) (Fig. 4b). Median survival for no treatment was 27,5 days, for MTX 30 µM 29,5 days and for MTX 120 µM 32 days.

Since we found a small treatment effect by MTX, we next investigated whether we could observe any changes in tumor size after treatment with 120 µM MTX in the SB28^CD24high^ model. In addition to untreated animals, we also included mice with pumps containing only saline, to rule out a purely mechanical influence of the pump. All mice were sacrificed on day 19 after tumor inoculation. Tumors were excised, serially sectioned, and stained with DAPI. Differences in tumor size among the no treatment group (*n* = 7), saline pump group (*n* = 7) and the group receiving a pump with 120µM MTX (*n* = 7) were analyzed using computerized image analysis. Three sections per tumor were included. Treatment with 120 µM MTX resulted in a significant reduction in tumor size compared to both untreated controls and saline-treated animals. Notably, tumors in the saline-treated group were larger than those in the untreated controls. Statistical analysis showed significant differences: 120 µM MTX *versus* untreated (*p* < 0,001****), 120 µM MTX *versus* saline (*p* < 0,001****), and untreated *versus* saline (*p* < 0,001****), showing that MTX effectively reduced tumor size in the SB28^CD24high^ model (Fig. 4c). Representative pictures of tumors from the different groups are shown in Fig. 4d.

In conclusion, a small effect on survival time as well as a reduced tumor size was recorded after intra-tumoral MTX treatment in SB28^CD24high^ mouse gliomas.

Analysis of CD24 expression of GL261- and SB28 mouse glioma cells using flow cytometry. GL261 (7,7%), SB28 (89,4%). Matched isotype antibodies were used as controls.4b. Kaplan Meyer survival curves of mice bearing established intracranial SB28^CD24high^ gliomas. MTX was administered using a mini-osmotic pump and brain infusion kit between days 7–13 for SB28 (dotted line). Concentrations used; 30 µM MTX (SB28 *n* = 8) 120 µM MTX (SB28 *n* = 17). Survival was compared to animals without any treatment (SB28 *n* = 28) as controls. A log-rank test was used to determine the difference between the MTX-treated mice (green line, blue line) and control mice without any treatment (black line). A *p*-value of < 0.05 was considered statistically significant. Survival was monitored for 100 days.

4c. For the tumor size study, mice were sacrificed on day 19 after tumor inoculation. The groups included: 120 µM MTX (*n* = 7), untreated mice (*n* = 7), and mice with pumps containing saline (*n* = 7). Tumor sections were stained with DAPI, and overviews were aquired at 10x magnification. Computerized image analysis was performed to calculate tumor area, expressed in mm^2^. Three sections per tumor were analyzed. Statistical differences were assessed using the Mann-Whitney *U*-test in Prism9^®^ (GraphPad Software, USA). A *p*-value of < 0.05 was considered statistically significant.

4d. Representative images of DAPI-stained tumors from the no-treatment, saline pump and MTX (120µM) groups are shown.

### Siglec-10^+^ cells interact with CD24 expressing SB28 mouse gliomas

**Fig. 5:**
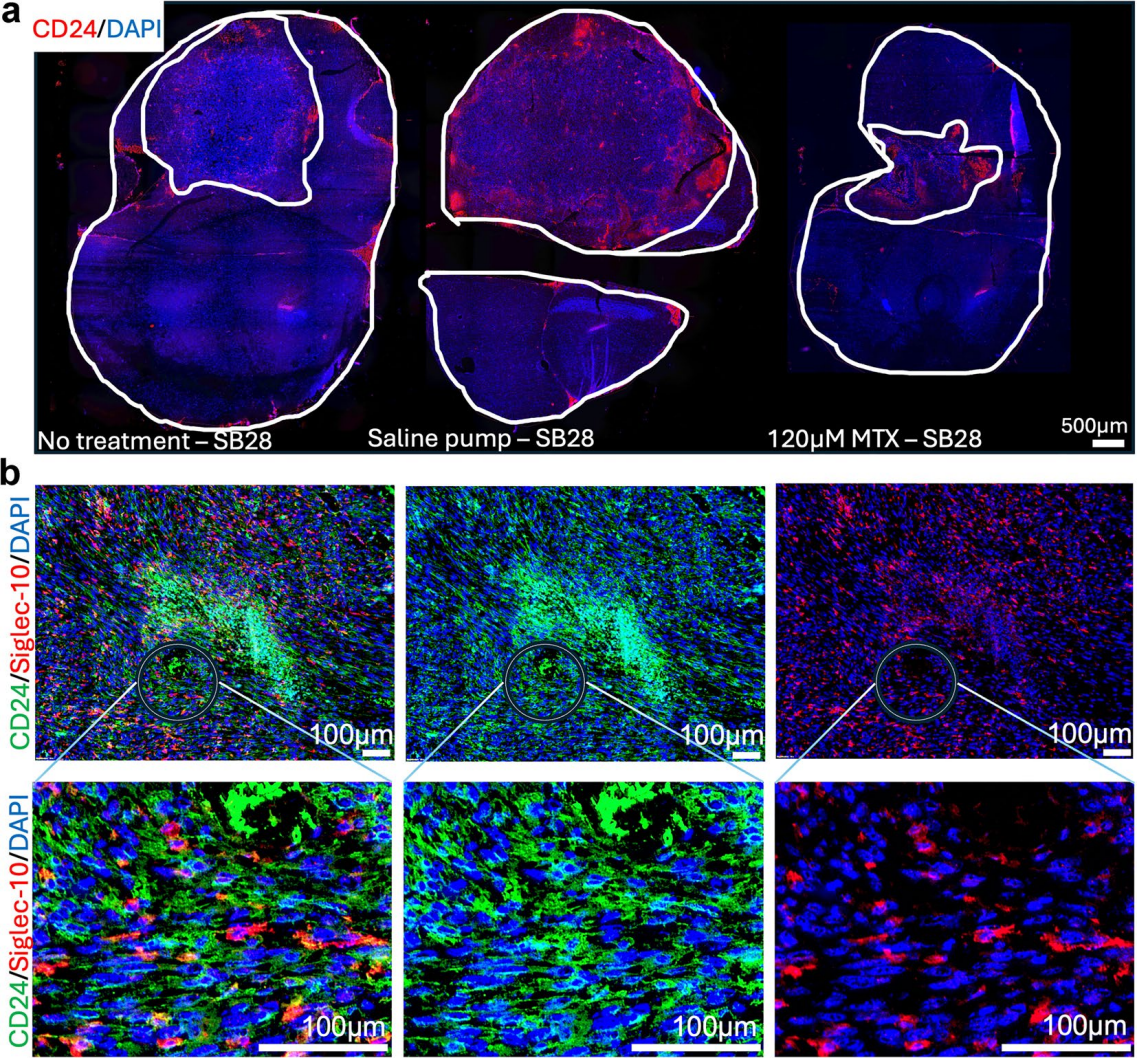
Siglec-10^+^ cells interact with CD24 expressing SB28 mouse gliomas.

In murine models, CD24 is expressed not only by tumor cells but also by erythrocytes and diverse immune cell populations^22,46^. This broad expression profile requires careful interpretation when assessing CD24 expression in tumors in vivo. Representative spatial overviews of CD24 distribution in SB28 gliomas and corresponding tumor sizes are depicted in Fig. 5a. Figure 5b illustrates CD24 (green) and Siglec-10 (red) localization within SB28 tumors, with the circle highlighting enlarged areas below. The enlarged image shows vesicular CD24 patterns on cells interacting with Siglec-10^+^ cells. Based on our observations, tumor cell CD24 expression can be distinguished with reasonable confidence by its characteristic vesicular pattern, in contrast to bone marrow-derived cells. To validate this, we co-stained CD24 with the pan- immune marker CD45 in SB28^CD24high^ and GL261^CD24low^ tumors (see supplementary Fig. S5). CD24^/^CD45 double-positive cells exhibited immune-like morphology in both tumors, whereas CD24^+^/CD45^-^ cells with vesicular CD24 staining were predominantly observed in SB28 tumors. This pattern was further confirmed in cultured SB28 cells (supplementary Fig. S4). In summary, although the widespread expression of CD24 in murine tumors limited direct assessment of drug-induced CD24 modulation, we verified that CD24^+^ tumor cells interact with Siglec-10^+^ cells in SB28 gliomas, mirroring interactions seen in human brain tumors (Fig. 3.)

Double-staining of SB28 mouse glioma tumor sections (immunohistochemistry). Sections from the tumor size study in Fig. 4.

- Representative spatial overviews (10x magnification) of mice receiving no treatment, saline pumps and intra-tumoral delivery of 120 µM MTX. CD24 (red). DAPI was used as nuclear staining (blue). White lines indicate tumor area, compared to total brain area. Scale bar, 500 μm.
- Representative image on the left shows the distribution of CD24 (green) and Siglec-10 (red) in the tumor microenvironment of SB28 mouse gliomas. The middle image shows CD24 only and image to the right shows Siglec-10 only. Enlarged images below, corresponding to areas marked with circles, display vesicular CD24 expression by SB28 tumor cells (green) interacting with Siglec-10^+^ cells (red). DAPI was used as nuclear staining (blue). Scale bar: 100 μm (10x magnification).

### MTX reduced Siglec-10^+^- and TREM2^+^-cells and increased CD8^+^-cells in SB28^*CD24high*^ tumors

**Fig. 6:**
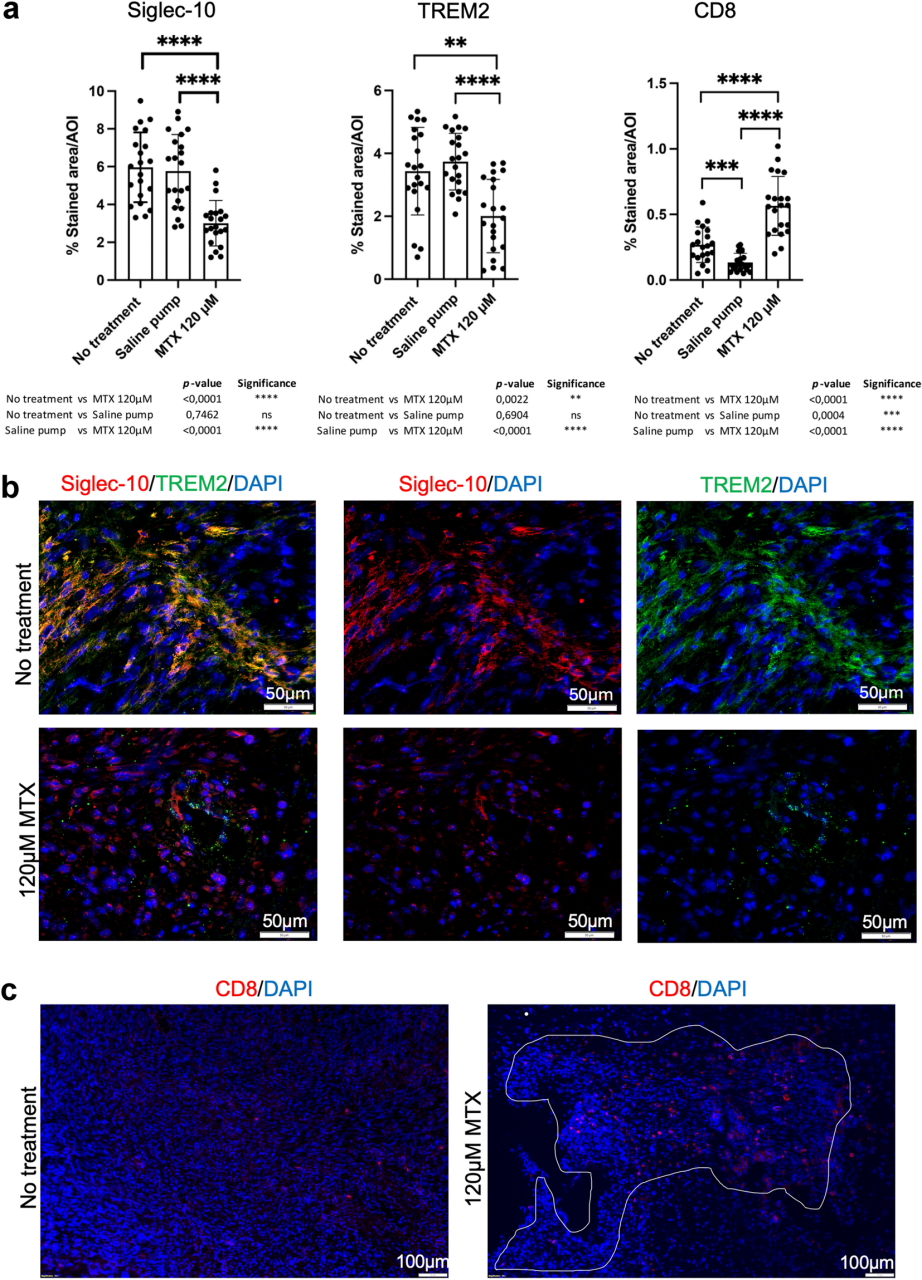
MTX-induced modulation of Siglec-10, TREM2 and CD8 in SB28 mouse gliomas.

Tumors included in the tumor size study were stained for Siglec-10, TREM2, and CD8 and analyzed for drug-induced differences. The ratio between Siglec-10, TREM2 or CD8 stained surface area compared to DAPI labeled tumor area was calculated. The stained area corresponding to Siglec-10^+^ cells was significantly decreased in MTX-treated animals (*n* = 7, 3 sections per animal) as compared to both no treatment (*n* = 7, 3 sections per animals) and to saline (*n* = 7, 3 sections per animal) (no treatment *versus* MTX *p* = < 0.0001****; saline *versus* MTX *p* = < 0.0001****; no treatment v*ersus* saline *p* = 0,7462 ns) (Fig. 6a). The stained area corresponding to TREM2^+^ cells significantly decreased in MTX-treated animals (*n* = 7, 3 sections per animal) as compared to no treatment (*n* = 7) and to saline (*n* = 7). There was no difference between no treatment and saline (no treatment *versus* MTX *p* = 0.0022**; saline v*ersus* MTX *p* < 0.0001****; no treatment *versus* saline *p* = 0.6904 ns) (Fig. 6a). The stained area corresponding to CD8^+^ cells was significantly increased in MTX-treated animals (*n* = 7, 3 sections per animal) as compared to both no treatment (*n* = 7, 3 sections per animal) and to saline s (*n* = 7, 3 sections per animal). CD8 + cells was also increased in no treatment compared to saline (no treatment *versus* MTX *p* < 0.0001****; saline *versus* MTX *p* < 0.0001****; no treatment *versus* saline *p* = 0.0004***) (Fig. 6a). Representative images of Siglec-10 and TREM2 double-staining in tumors from non-treated animals and in MTX-treated animals showed that Siglec-10 and TREM2 co-localized and that both markers were reduced after treatment (Fig. 6b). In addition, representative pictures of CD8 in tumors from non-treated animals and in MTX-treated animals showed that CD8-labeling was increased after treatment (Fig. 6c).

**6a.** Tumor sections included in the tumor size study were stained for Siglec-10, TREM2 or CD8. DAPI was used as nuclear staining. Overviews were taken at 10x magnification. Computerized image analysis was performed and the ratio between the Siglec-10, TREM2- or CD8-stained area compared to DAPI-stained tumor area was calculated. Seven animals per group and 3 serial sections per tumor were analyzed. Differences were calculated with the Mann-Whitney *U*-test using Prism9^®^ software (GraphPad software, USA). A *p*-value of < 0.05 was considered statistically significant. **6b.** Double-staining of Siglec-10 (red) and TREM2 (green) in frozen SB28 tumors. DAPI was used as nuclear staining. Scale bar 50 μm (20x magnification). **6c.** Staining of CD8 (red) in SB28 tumors. Picture to the left, SB28 tumors without treatment covers the whole picture area. Picture to the right, white marking, indicates the smaller tumor area after treatment with 120 µM MTX. Scale bar 100 μm (10x magnification).

### MTX induces a dose dependent cell death and modulates CD24 expression

**Fig. 7:**
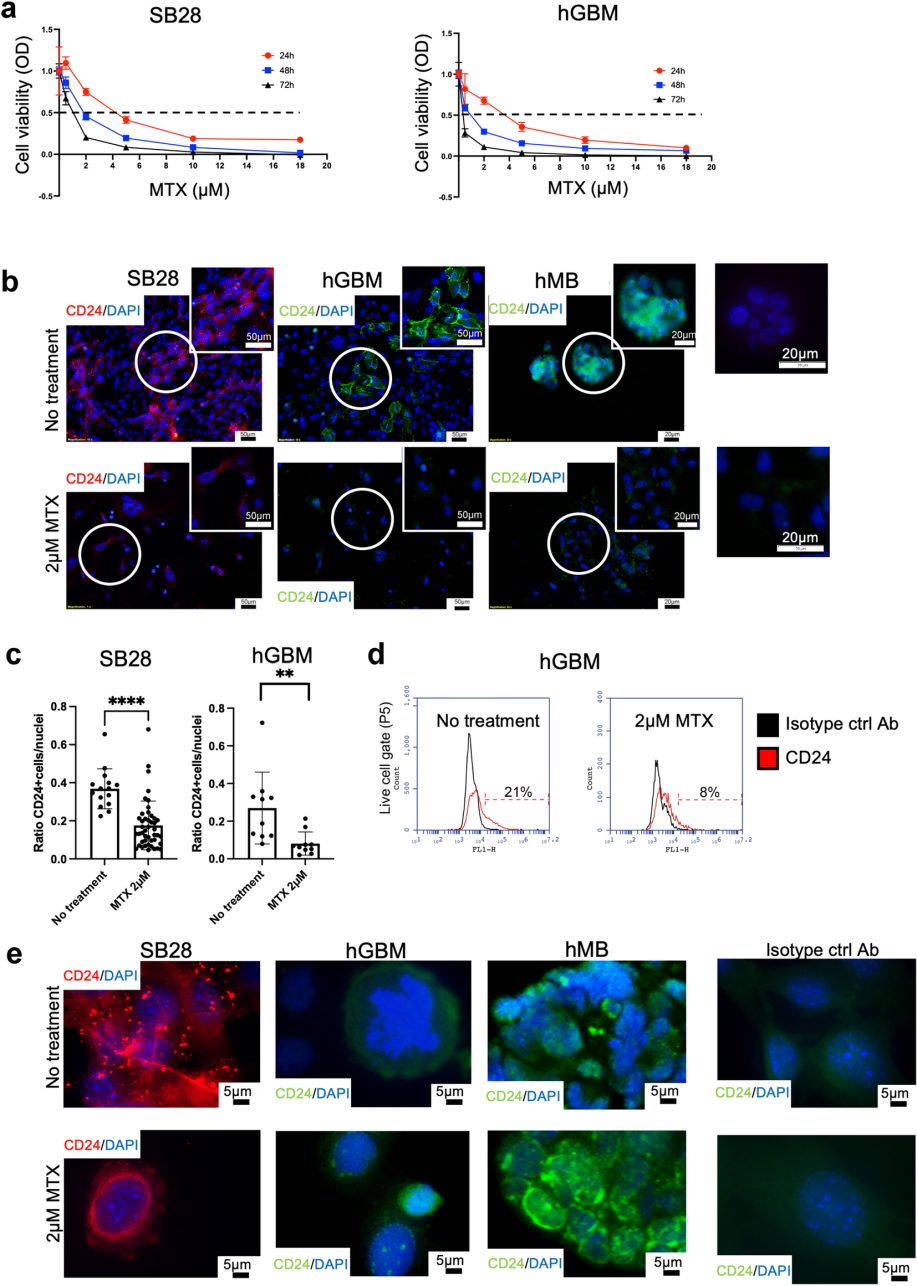
MTX-induced changes in cell viability and CD24 expression.

To verify the direct cytotoxic effects of MTX on the murine SB28 cell line, as well as on hGBM, we subjected cell cultures to treatment with increasing doses of the drug (0.5, 1, 2, 5, and 18 µM) for 24, 48, and 72 h. SB28 cells were less sensitive than hGBM cells, but 10µM MTX caused > 90% of all cells to die (Fig. 7a). Cell death of 50% of SB28 cells was reached after 48 h at 2 µM MTX-treatment, therefore this dose was chosen in the next experiment. SB28 mouse glioma cells express high levels of CD24 as determined by immunocytochemistry of cultured cells, while we have shown in previous publications that cultured hMB also express high levels of CD24^33^. Hence, we investigated whether MTX modulated CD24 expression by treating tumor cells with 2 µM MTX. Immunocytochemical staining of SB28, hGBM, and hMB cells showed that MTX reduced the expression level of CD24 on all tumor cells investigated, as compared to non-treated cells (Fig. 7b). Computerized image analysis of SB28 (*p*-value < 0.0001****) and hGBM cells (*p*-value 0.0011**) showed a significant reduction of CD24 expression after MTX-treatment (Fig. 7c). Flow cytometric cytometric analysis of hGBM cells confirmed this (hGBM no treatment 21% CD24, hGBM 2 µM MTX 8% CD24) (Fig. 7d). To investigate changes in intracellular CD24 expression following MTX treatment, we applied an alternative staining protocol (see Materials and Methods). Qualitative alterations in CD24 expression were observed in all analyzed cells. After MTX treatment, CD24 localized around the nuclei and appeared in larger clusters compared to untreated cells. In SB28 cells, the staining pattern suggested a loss of the vesicular distribution of CD24 following MTX exposure (Fig. 7e).

**7a.** Cell viability was investigated using an MTT-assay. Adherent cells (SB28, hGBM) were treated with 0.5, 1, 2, 5, 10, or 18 µM of MTX for 24, 48, and 72 h. OD, optical density. Dotted line indicates 50% cell death. **7b.** Cells cultured in multi-chamber slides and stained for CD24; SB28 (red), hGBM (green), and hMB (green), using immunocytochemistry. Upper panel: no treatment. Lower panel: 2µM MTX for 48 h. Representative images from one experiment are shown. DAPI was used as nuclear staining. Scale bar 50 μm (20x magnification) or 20 μm (40x magnification). Matched isotype control antibodies are shown to the right. **7c.** Cells (SB28, hGBM) were cultured in multi-chamber slides, treated with 2µM MTX for 48 h and stained for CD24. Pictures were taken at 10x magnification for image analysis. Amount CD24-labeling per picture was analyzed and differences were calculated with the Mann-Whitney *U*-test using Prism9^®^ software (GraphPad software, USA). A *p*-value of < 0.05 was considered statistically significant. **7d.** hGBM cells were treated with 2 µM of MTX for 48 h and changes in CD24 expression were analyzed with flow cytometry. Histogram shows a change from 21% CD24^+^ cells to 8% CD24^+^ cells after treatment. Matched isotype antibodies were used as controls. **7e.** Cells (SB28, hGBM hMB) were cultured in multi-chamber slides, treated with MTX for 24 h and stained for intra-cellular CD24. Untreated cells were used as controls. Matched isotype control antibodies are shown to the right. Representative images are shown. DAPI was used as nuclear staining. Scale bar 5 μm (100x magnification).

## Discussion

Several studies have reported that high expression of CD24 or Siglec-10 is associated with poor survival in gliomas, including GBM, and in some MB subgroups^32,47^. However, these associations are not consistent across all datasets or tumor subtypes, and prognostic significance varies depending on molecular context and cohort size. Importantly, even if CD24/Siglec-10 interaction does not universally predict outcome, it remains a biologically relevant immune checkpoint. Disrupting this axis could restore innate immune clearance and improve therapeutic response, independent of its prognostic value.

CD24 plays a role in promoting stemness, proliferation, malignancy, and metastasis, as well as suppressing anti-tumor immunity, underscoring its potential as therapeutic target^19,42–45^. However, CD24 is expressed not only by brain tumor cells but also by various normal cell types, including nervous system cells, immune cells^22^, and even erythrocytes in mice^46^. This broad expression complicates efforts to selectively target tumor-associated CD24 without affecting normal tissues. Additionally, the dual role of intracellular CD24—ranging from promoting drug resistance to inhibiting tumor cell invasion—further complicates therapeutic targeting^48,49^.

We and others have identified subgroup-specific expression patterns of CD24 and Siglec-10 in brain tumors which also needs to be considered when developing treatment modalities against the CD24/Siglec-10 axis^32–34^. The heterogeneous expression of CD24 in GBM compared to MB, along with the distinct vesicular patterns observed in MB, suggests that tumor- as well as subgroup-specific targeting approaches may be required. *CD24* correlates with genes associated with unfavorable prognostic features in SHH and Group4 MB as well as in IDHmut GBM tumors, supporting the potential benefit of targeting CD24 in these subtypes. High risk IDHmut tumors display increased cell division, DNA replication and repair, mitotic cell cycle transition, and p53 signaling pathway activity^50^, reinforcing the rationale for CD24-directed therapy.

Although GBM and MB are considered immunologically cold tumors, single-cell RNA-seq and transcriptomic studies show that MG and TAMs are present and significantly influence tumor biology. Even modest changes in TAM/MG abundance or phenotype can impact prognosis, as enrichment of immunosuppressive subsets correlates with worse outcomes in GBM^51^. Treatment modalities may also influence dynamic TAM populations in SHH MBs^52^.

In this study, we demonstrate that Siglec-10^+^ cells represent MG-like cells in both MB and GBM, as evidenced by both gene expression profiles and tissue section analyses. Siglec-10 and TREM2 were co-expressed on cells with long protrusions, and Siglec-10 was more closely associated with gene profiles characteristic of pro-tumoral M2 macrophages. Notably, the SHH subgroup was unique in exhibiting a higher M1 than M2 profile. Subgroup-specific TAM differences could impact response, highlighting the need for biomarkers to guide immunotherapy.

Our analysis of MB gene expression data revealed consistent results across different cohorts, whereas GBM data were more heterogeneous. Gene expression profiles for GBM^PN^ tumors from the Rembrandt dataset resembled those of IDH-mutant tumors, suggesting that many GBM^PN^ tumors in this cohort may actually be IDH-mutant.

The SB28 mouse glioma model is increasingly recognized for its suitability in immunotherapeutic studies, primarily due to its lower immunogenicity and fewer mutations compared to the more commonly used GL261 mouse glioma model^53^. SB28 cells exhibited very high levels of CD24, mirroring the expression pattern seen in hMBs. CD24^+^ cells with a vesicular staining pattern interacted with Siglec-10^+^/TREM2^+^ cells in tumors, underscoring the relevance of this model for targeting the CD24/Siglec-10 axis.

One mechanism by which CD24 and Siglec-10 may contribute to immune evasion is through suppression of immune responses triggered by cellular damage, mediated by HMGB1 – a key marker of immunogenic cell death (ICD)^17^. MTX has been shown to induce ICD, characterized by the release of danger-associated molecular patterns (DAMPs) that activate dendritic cells and promote T-cell priming^54^, suggesting that MTX could also affect the CD24/Siglec-10 interaction. Similarly, doxorubicin reduces CD24 expression in triple-negative breast cancer cells, and CD24-overexpressing cells are more sensitive to doxorubicin treatment, partly through modulation of autophagy^55^. As MTX is a synthetic derivate of doxorubicin, it may exert comparable effects on CD24-high tumor cells. Autophagy is a key event in MTX-induced cell death^56^, and its relationship with CD24-modulation warrants further investigation^57^.

MTX was delivered locally into the tumor using mini-osmotic pumps, which minimizes systemic toxicity and ensures sustained drug exposure within the tumor microenvironment. This localized delivery is particularly advantageous in brain tumors, where systemic administration often fails to achieve therapeutic concentrations without severe side effects.

Local delivery of the poorly lipid-soluble substance MTX to brain tumors has been tested in clinical trials in conjunction with the standard drug, Temozolomide^29,30^. New and potentially more efficient methods of delivering drugs directly to tumors, including brain tumors, involve the development of MTX-containing nanoparticles and the intra-arterial route of administration^58,59^.

Intratumoral MTX delivery produced a small but statistically significant survival benefit and reduced tumor size in SB28^CD24high^ gliomas, accompanied by increasing CD8⁺ cytotoxic T-cell infiltration and reducing immunosuppressive populations such as Siglec-10⁺ and TREM2⁺ cells. Additionally, MTX showed dose-dependent effects on tumor cells, including decreased viability and altered CD24-expression, underscoring its potential to target CD24 and it´s interaction with Siglec-10. Given that TREM-inhibition enhances checkpoint immunotherapy^40^, our data suggest that TREM-targeting may further influence this axis.

In summary, targeting the CD24/Siglec-10 axis may be of interest, particularly in SHH medulloblastomas, GBM^PN^, and IDHmut GBM tumors, where CD24 expression on tumor cells and Siglec-10 + cells’ TAM-profile suggest a more favorable therapeutic opportunity. The small but significant effects of MTX in the therapy-resistant SB28 mouse glioma model highlight the potential of targeting this axis. Studies of several pathological entities where blocking of CD24/Siglec-10 by antibodies or small molecules was investigated as monotherapy, show modest outcome improvement but is substantially potentiated by simultaneous blockage of other immunosuppressive mechanisms^18–20,60^ and the multifactorial nature of immunosuppression in MB and GBM suggests that combination therapies may be necessary.

Further research and clinical trials are needed to refine these strategies and fully exploit the therapeutic potential of CD24/Siglec-10 targeting in brain tumors.

## Materials and methods

### Gene expression data

Normalized gene expression from the Cavalli MB dataset (WNTα *n* = 49, WNTβ *n* = 21, WNT *n* = 70, SHHα *n* = 65, SHHβ *n* = 35, SHHγ *n* = 47, SHHδ *n* = 76, SHH *n* = 223, Group3α *n* = 67, Group3β *n* = 37, Group3γ *n* = 40, Group3 *n* = 144, Group4α *n* = 98, Group4β *n* = 109, Group4γ *n* = 119, Group4 *n* = 326) including patients over 18 years, and from the Northcott_2012 MB dataset (SHH *n* = 51, Group3 *n* = 46, Group4 *n* = 188) were used^8,61^. Normalized gene expression from the TCGA_GBM dataset (TCGA Research Network^62^; HG-U133A (Classical (C) *n* = 199, Mesenchymal (M) *n* = 166, Proneural (PN) *n* = 163, IDHwt *n* = 372, IDHmut *n* = 30); Agilent 4502 A (C *n* = 182, M *n* = 156, PN *n* = 151, IDHwt *n* = 339, IDHmut *n* = 27) and from the Rembrandt dataset was used (C *n* = 79, M *n* = 70, PN *n* = 70), excluding normal tissue. Gene expression data was retrieved from the GlioVis data portal except for *CD24* gene expression under the Cavalli dataset that was retrieved from R2: Genomics Analysis and Visualization Platform http://r2.amc.nl (Cavalli, CD24-reporter 8177222). For GBM, IDHwt and IDHmut, *CD24* and genes correlated with *CD24* was retrieved from the TCGA_GBM (platform HG-U133A). For GBM, IDHwt and IDHmut, *SIGLEC-10* and genes correlated with *SIGLEC-10* was retrieved from the TCGA_GBM (platform Agilent 4502 A).

A Spearman correlation test was used to investigate the association between genes. The ratio for the gene of interest (*CD24*,* MKI67*, or WNT-*CTNNB1*, SHH-*SMO*, Group3-*MYC*, Group4-*CDK6* for MB) was calculated by dividing the number of correlated genes (including weak, moderate, and high correlations) per gene of interest by 10. The 10-gene signature represents the following genes:

- **Positive correlation high-risk**: *FOXM1*,* NEK2*,* CCT2*,* ACTL6A*,* CCND2*,* ABL1*,* SYNCRIP*,* ITGB*,* ENAH*,* UMPS*.
- **Negative correlation low-risk**: *ADAM22*,* AEBP1*,* DHRS2*,* RAC3*,* SHANK1*, CYB5D2*,* IL27RA*,* DNAH2*, ZPF3*,* NRXN2*.

Genes marked with an asterisk (*) were not available in the TCGA_GBM dataset, and the ratio was calculated by dividing by 8.

The ratio for the gene of interest *(SIGLEC-10*,* TREM2*,* CD163*, or *ITGA4*) was calculated by dividing the number of correlated genes (including weak, moderate, and high correlations) by 12 per gene of interest. The 12-gene signature represents the TAM-phenotypes:

- **M1**: *APOL3*,* CCL19*,* CCL5*,* CCR7*,* CD38*,* CD40*,* CXCL10*,* CXCL9*,* EBI3*,* IDO1*,* LAMP3*,* TNFAIP6*.
- **M2**: *AIF1*,* CCL13*,* CCL18*,* CD180*,* CD209*,* CD4*,* CLEC4A*,* CLEC10A*,* MS4A6A*,* NPL*,* SLC15A3*,* TREM2*.

Genes representing M1 and M2 TAMs were selected based on defined expression profiles according to the CIBERSORT analytical tool. For the TCGA_GBM, IDHwt, and IDHmut tumors, *IDO1* was not present in the dataset, and the ratio was calculated by dividing by 11. When *TREM2* was selected as the gene of interest, the ratio for the M2-profile was calculated by dividing by 11, as *TREM2* is contained within the M2-phenotype.

A Spearman r-value equal to or above 0.26 and a p-value < 0.001 was considered a weak positive correlation. A Spearman r equal to 0.30 or above and a p-value < 0.05 was considered a moderate positive correlation. A Spearman r equal to 0.50 or above and a p-value < 0.05 was considered a strong positive correlation. A Spearman r equal to or below − 0.26 and a p-value < 0.001 was considered a weak negative correlation. A Spearman r equal to −0.30 or below and a p-value < 0.05 was considered a moderate negative correlation. A Spearman r equal to −0.50 or below and a p-value < 0.05 was considered a strong negative correlation.

### Subtyping of biobanked tumor tissue

To classify biobanked GBM tumor tissue into transcriptional subtypes, normalized expression data from snRNA-seq of human GBMs (GSE237673) were entered into GSEA software (Broad Institute/UC San Diego, version 4.1.0) as sample expression data for the GseaPreranked tool. Established gene signatures for GBM subtypes (Classical, Proneural, and Mesenchymal) defined by Verhaak et al.^5^ were retrieved from the Molecular Signatures Database (MSigDB) (https://www.gsea-msigdb.org/) and used as genesets. Chip platform utilized was Human_ENSEMBL_Gene_ID_MSigDB.v7.4.chip; maximum set size was set to 1000; mode chosen was Max_probe; and the collapsing parameter was set to Collapse. All other parameters were maintained according to the program’s default settings. The GBM subtype associated with the highest Normalized Enrichment Score (NES) was designated as the most likely subtype for each sample.

MB-subtyping is performed within clinical routine pathology, and data was obtained from the patient record.

### Ethical permits

Human cells and tissue were obtained from Region Skåne biobank, BD 27 Dnr: 2018/37 under ethical permit Dnr:2021/05184, approved by Regional Ethics Review Board in Lund, Sweden and Swedish Ethical Review Authority, and all patients and/or their parents gave their written informed consent prior to inclusion in the study. All sections of this study involving human participants are performed in accordance with the Declaration of Helsinki (https://www.wma.net/policies-post/wma-declaration-of-helsinki-ethical-principles-for-medical-research-involving-human-subjects/). The SB28 mouse glioma cell line (SB28 – luciferase^+^/GFP^+^), syngeneic with the C57BL/6 strain, was kindly provided by Dr. Mats Hellström, Uppsala University, Sweden with permission from Dr. Hideho Okada, UCSF, USA. Animal experiments followed the Swedish Board of Animal Research and European Union Animal Rights and Ethics Directives and were approved by the Ethical Committee of Animal Ethics in Lund-Malmö (14006/2019). All sections of this study adhere to the ARRIVE Guidelines for reporting animal research^63^.

### Cell lines and cell culture medium

Mouse glioma cells (GL261, SB28) and hGBM cells were cultured at 37 °C in the presence of 5% CO_2_ in R10-medium containing: RPMI 1640 medium supplemented with 2 mM L-glutamine, 1 mM sodium pyruvate, 10mM HEPES, 50 µg/mL gentamicin (GIBCO, fisher scientific, Sweden) and 10% fetal bovine serum (BIOWEST, VWR, Sweden). Adherent cells were rinsed with PBS and detached using TrypLE^®^Express (GIBCO). hMB cells were cultured in serum free cell culture medium (UltraCULTURE™, Lonza BioWhitaker Inc., VWR, Sweden supplemented with 2mM L-glutamine, 1% Penicillin-Streptomycin, Life Technologies, or with NeuroCult™ NS-A Proliferation kit (human), STEMCELL-technologies, Europe) supplemented with 50 µg/mL gentamicin (GIBCO) with the addition of EGF 20 ng/ml, and bFGF 20–40 ng/ml (Chemicon, Merck Millipore, Sweden) in cell culture flasks without adherence (UltraLow™, Corning, Saveen & Werner AB, Sweden). For tumor inoculation of SB28 cells, fetal bovine serum and gentamicin were excluded in the medium and referred to as R0-medium.

### Experimental animals

C57BL/6 female mice 8–10 weeks old were purchased from Taconic, Denmark and maintained in specific pathogen-free conditions in a temperature- and humidity-controlled environment (21° ± 1°, 55% of humidity) with 11-hr light/13-hr dark cycle at BMC, Lund University, Lund, Sweden. Upon arrival, mice carried their respective health monitoring report and were given five days to acclimate to the housing facility. Then, animals were housed together in groups of five in Innocage^®^ cages (pre-bedded with corn cob, one sheet of Innorichment^™^ and one 10 × 10 × 50 mm aspen chew block, Innovive, USA) with constant access to food (RM3(P) pellets, SDS diet, England) and water.

### Experimental design of in vivo studies

We aimed to evaluate the effect of intratumoral MTX in the experimental mouse glioma model SB28. Mice were randomized for in vivo experiments, into the corresponding intervention group and the control groups as well as prior to interventional procedures whereas data analysis was performed without blinding. Following groups were included in the survival experiment (1) mice receiving MTX via a mini-osmotic pump (30µM *n* = 8), (2) mice receiving MTX via a mini-osmotic pump (120µM *n* = 17), and (3) mice without pumps (*n* = 28). The mice were carefully observed daily for signs of drug toxicity, such as seizures and for neurological symptoms due to tumor growth. When neurological symptoms appeared, mice were immediately euthanized. All brains were examined for macroscopic evidence of tumor growth.

For the tumor size and immune cell infiltration study, following groups were included (1) mice receiving MTX via a mini-osmotic pump (120µM *n* = 7), (2) mice without pumps (*n* = 7) and (3) mice with pumps containing saline (*n* = 7). In this study, animals were sacrificed 19 days after tumor challenge. All brains were examined for macroscopic evidence of tumor growth.

### MTX and Preparation of micro-osmotic pumps

The chemotherapeutic agent MTX (mitoxantrone, 2 mg/ml Ebewe, Sweden) was used for all in vitro and in vivo experiments. MTX solution was diluted in sterile PBS to the appropriate concentrations. For intra-tumoral delivery of MTX or for delivery of sterile saline (0.9% NaCl) in the SB28-model, 7-day micro-osmotic pumps Alzet^®^ model 1007D (fill volume 100 µl, pumping rate 0,5 µl/h; DURECT Corporation) were used. The pumps were filled with MTX (30 or 120 µM) or sterile saline (0.9% NaCl) and coupled to the Alzet^®^ brain infusion kit 3 (Nova SCB AB, AgnThos´, Sweden) with a 2.5 cm catheter tube according to the manufacturer’s protocol. The assembled pumps were incubated at 37 °C overnight in sterile PBS before use.

### Brain tumor model and drug-delivery via pumps

On day 0 brain tumors were induced by inoculation of 1600 SB28 tumor cells into the right frontal lobe. Cells were contained in R0 culture medium, see above. Mice were anesthetized with 2% Isoflurane Forene^®^ (Abbott Scandinavia AB) delivered in pure O_2_ (200 ml/s), fixed and immobilized in a stereotactic frame (Kopf Instruments). The scalp was disinfected with 70% alcohol and 0.05 ml Marcaine^®^ (Bupivacaine hydrochloride 2.5 mg/ml + epinephrine 5 µg/ml) was injected subcutaneously. A linear skin incision starting in the midline between the eyes and ending in the midline shortly behind the bregma was performed. A small hole was drilled into the skull with a 0.5 mm rose-head drill bit 1.5 mm to the right and 1.0 mm anterior of the bregma. A Hamilton syringe (Hamilton, AgnThos´, Sweden) with a 33 G blunt needle was used to inject 3 µl of cell suspension 2.75 mm deep from the dural surface. The cell suspension was delivered slowly over the course of 5 min. Following injection, the needle was left in place for 3 min, then raised to a depth of 1.5 mm below the brain surface and left in place for an additional minute to diminish any backflow through the canal. Upon withdrawal of the needle, the burr hole was sealed with bone wax and the incision was closed with one 7.5 mm metal clip.

For the micro-osmotic pump implantation, tumor-bearing mice were anesthetized and fixed as described above. The previous skin incision was re-opened and the pump assembly was implanted into a subcutaneous pocket in the midscapular area. Subsequently, the brain infusion kit was inserted through the original hole in the skull and fixed to the skull with cyanoacrylate adhesive Alzet-LOCTITE^®^ gel (DURECT Corporation). Finally, the incision was closed with one 7.5 mm metal clip. The pumps were removed after the designated working time elapsed. Kaplan-Meier survival curves were compared using a log rank Mantel-Cox test. A *p*-value < 0.05 was considered statistically significant. All statistical analyses were performed using Prism9^®^ software (GraphPad software, USA).

### Immunohistochemistry of frozen tumor sections

Brain tissue was frozen and fixed in cooled isopentane (−55 °C, VWR, Sweden) and kept thereafter at −80 °C until further analysis. Before staining, brain tissue was sectioned into 6 μm-thick sections on a cryostat (CryoStar NX50, Epredia, Cellab, Sweden). Then, sections were fixed with acetone for 10 min. at room temperature, rehydrated with PBS without Ca^2+^ and Mg^2+^ (Gibco), blocked for 20 min with 5% goat serum (Jackson ImmunoResearch, USA) and single stained or double stained for 60 min. with following antibodies; rat-anti mouse CD24 clone M1/69 cat#14–0242-85; rat anti-mouse CD163 cat#14–1631-82 (eBioscience™, Invitrogen, Sweden); CD24 clone ML5-FITC cat#60,992; mouse-anti human FITC-CD163 cat#563,697; rat-anti mouse CD8α cat#550,281 (BD Biosciences, BD Pharmingen™, Sweden); rabbit-anti mouse/human Siglec-10 cat# BS-2707-R (ThermoScientific, Sweden); rat anti mouse/human TREM2 cat#MAB17291 (R&D systems, Bio-Techne). Sections were incubated for 30 min. with the following secondary antibodies; goat anti-rat Alexa 488 and 594, goat anti-mouse Alexa 488, goat anti-rabbit 594 (MolecularProbes™, Invitrogen, Sweden). Matched isotype antibodies were used as controls. PBS were used to rinse the sections between the different steps followed by mounting in DAPI-containing mounting medium (ProLong™ Gold antifade, Invitrogen, Sweden). To cover the whole brain tumor section, spatial overviews were performed at 10x magnification using the Cellsens Dimension software on the fluorescent microscope BX-53 (both Olympus LRI Instrument, AB). Pictures were also taken at 10x, 20x and 40x magnification in the separate fluorescens-channels, and aligned pictures were created using the Cellsens Dimension software.

### Immunocytochemical staining of CD24 after exposure to MTX in vitro

Immunocytochemistry was used to evaluate the change in CD24 expression after exposure to 2 µM MTX of SB28 mouse glioma cells, hGBM and hMB cells. Cells were cultured for 24 h in 8-well chamber culture slides (BD Biosciences). To adhere MB spheres to the chamber slides, slides were pre-incubated with Poly-L lysine (Gibco) over night at room temperature before spheres were added to the wells. 2 µM MTX was added to the wells for 48 h. Cells were fixed in 4% paraformaldehyde for 30 min, and blocked with 5% goat serum diluted in PBS for 20 min. Cells were incubated with the primary antibody (rat-anti mouse CD24 clone M1/69 cat#14–0242-85, LifeTechnologies, eBioscience™, mouse anti human CD24 ML5-FITC cat#60992, BD Biosciences, BD Pharmingen™) for 2,5 h at 37 °C, followed by the secondary antibody, goat-anti rat Alexa 594, or goat-anti mouse Alexa 488 (Molecular Probes) for 30 min at RT. The chamber slides were mounted wet using Pro-Long Gold anti-fading reagent with nuclear DAPI staining (Molecular Probes). PBS was used in all washing steps and as a diluent for reagents. Images were acquired using an Olympus BX-53 fluorescent microscope (LRI instrument AB) at 10X magnification.

For intra-cellular staining of CD24, cells were fixed in 4% paraformaldehyde for 30 min, blocked with 5% goat serum diluted in Tween-20 0,5%/PBS (T-PBS) for 60 min. and permeabilized with PBS containing 10% Triton X100 for 30 min. Cells were incubated with the primary antibody over-night at 4 °C, followed by a secondary antibody incubation for 30 min. The same primary- and secondary antibodies as used above where used. The chamber slides were mounted wet using Pro-Long Gold anti-fading reagent with nuclear DAPI staining (Molecular Probes). PBS was used in all washing steps and as a diluent for reagents. Images were acquired using an Olympus BX-53 fluorescent microscope (LRI instrument AB) at 100X magnification.

### Flow cytometric measurement of CD24 expression

Flow cytometry was used to evaluate CD24 expression on cultured mouse glioma cells (GL261, SB28) and cultured human GBM cells. Cells were stained for rat-anti mouse CD24 or mouse-anti human CD24-FITC for 40 min at 4 °C. Secondary antibodies used were mouse-anti rat Alexa-488 and 594. Detailed description of antibodies, see above. Matched isotype control antibodies and unstained cells were used as negative controls. Cells were washed with PBS + 0.5% BSA between staining steps. Fluorescence staining intensity on mouse glioma cells was measured at the Multipark FACS core facility, BMC, Lund Sweden and analyzed using BD FACSDiva 8.0.1 software. Fluorescence staining intensity on hGBM cells were measured using an Accuri^®^ flow cytometer and analyzed using BD Accuri C6 software.

### Measurement of cell viability

Cell viability after MTX treatment was assessed by the MTT (3-(4,5-dimethylthiazol-2-yl)−2,5-diphenyltetrazolium bromide) assay (Sigma-Aldrich, Merck, Sweden). MTT (5 mg/ml) was dissolved in PBS and frozen at −20 °C. Adherent SB28 and hGBM cells were washed with PBS and detached with TrypLE^®^Express (GIBCO). 2 × 10^3^cells were seeded into 96 well plates (Corning 96 well culture plates, flat bottom with lid). MTX (mitoxantrone, 2 mg/ml Ebewe, Sweden) was diluted in R10 to the appropriate concentrations (no treatment, 0.5, 1, 2, 5, 10 or 18 µM) and cells were treated in 100 µl for 24, 48–72 h. Six wells per treatment were analyzed. MTT-solution (10 µl) was added to each well for 3 hours, followed by DMSO (100 µl) (Sigma-Aldrich). Optical density (OD) was analyzed at 540 nm and 650 nm using the plate reader (spectramax^®^ iD3; Molecular Devices, VWR, Sweden) and the spectramax^®^iD3 software. Values were normalized to untreated cells and cell viability curves were plotted using Prism9^®^ software (GraphPad software, USA). One representative experiment out of > 3 is shown.

### Computerized image analysis of tissue sections

Tumor sections from animals sacrificed on day 19 after SB28 tumor inoculation analyzed for tumor size and immune cell infiltration. The study included three groups: no treatment *n* = 7, saline pumps *n* = 7, 120 µM MTX *n* = 7, 3 with three sections analyzed per tumor.

Sections were single-stained for Siglec-10, TREM2 and CD8 and nuclei were stained with DAPI. Spatial overviews and tumor area measurements (mm^2^) were obtained at 10x magnification using the Cellsens Dimension software on the fluorescent microscope BX-53 (both Olympus LRI Instrument, AB). Immune cell infiltration was quantified by applying a pseudocolor to stained cells and calculating the ratio of stained area to tumor area. Statistical differences were assessed using the Mann-Whitney *U*-test in Prism9^®^ (GraphPad Software, USA). A *p*-value of < 0.05 was considered statistically significant.

### Computerized image analysis of cultured cells

Analysis of CD24 on cultured cells was analyzed on 10 or more images per well and treatment, using Cell Dimension software, Olympus (LRI instrument AB). The same intensity settings were used irrespective of treatment. The ratio between CD24 stained area and the number of DAPI stained nuclei was compared between groups (for SB28- and human GBM cells) with the Mann-Whitney *U*-test using Prism9^®^ software (GraphPad software, USA). *p* value of < 0.05 were considered statistically significant. One representative experiment out of > 3 is shown.

## Supplementary Information

Below is the link to the electronic supplementary material.

Supplementary Material 1

Supplementary Material 2

## Data Availability

Normalized expression data from snRNA-seq of human GBMs included in the study are deposited under GSE237673.
