# Supplementary material for: Mitoxantrone alters CD24/Siglec-10 expression in malignant brain tumor models
Source: Sci Rep. 2026 Jan 28;16:3696. doi: 10.1038/s41598-026-37588-7 (PMC12852714; doi:10.1038/s41598-026-37588-7)
Supplement: Supplementary file 1 — Supplementary Material 1 [file 41598_2026_37588_MOESM1_ESM.docx]

**Supplementary material:**


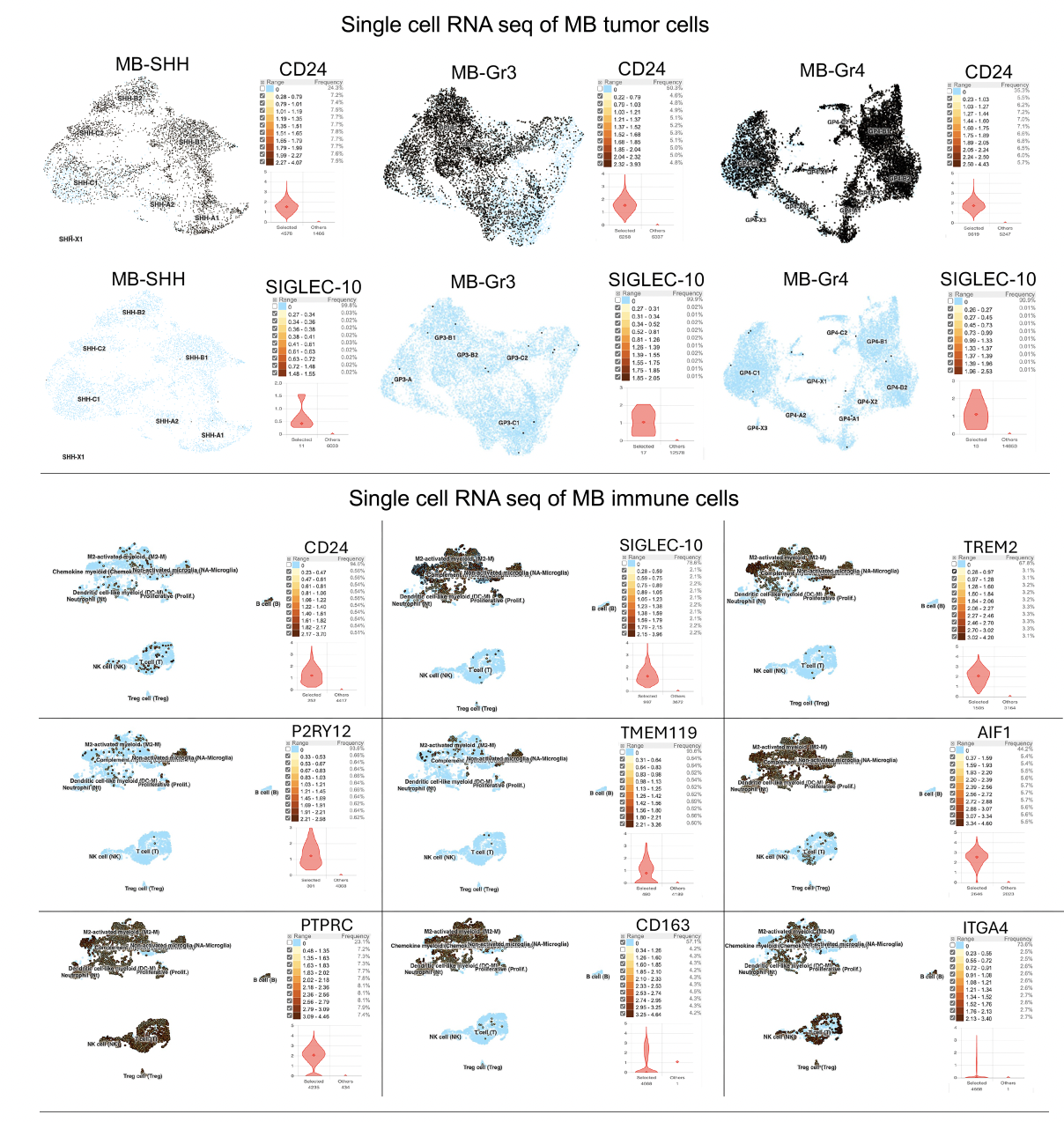


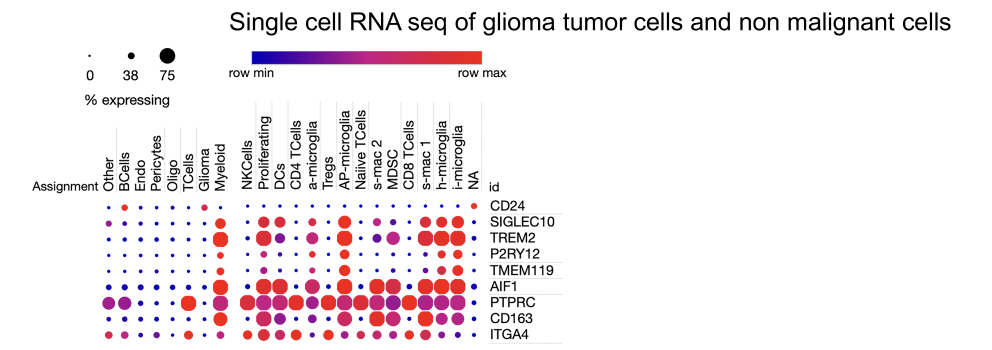


**Figure S1**. Single-cell RNA sequencing data

Upper panel: Single-cell RNA sequencing data showing *CD24* and *SIGLEC-10* expression in SHH, Group 3 and Group 4 MB tumor cells. Middle panel: Single cell RNA-sequencing data of MB immune cells for *CD24, SIGLEC-10, TREM2, P2RY12, TMEM119, AIF1, PTPRC* (CD45), *CD163* and *ITGA4* (CD49d). MB data was obtained from the interactive Pediatric Neuro-oncology Cell Atlas browser (pneuroonccellatlas.org), under the Single-cell RNA-seq of childhood medulloblastoma samples (n=28) originally published by Riemondy et al., GEO dataset GSE156053 [64]. Lower panel: Dot plot of single-cell RNA sequencing data showing expression of *CD24, SIGLEC-10, TREM2, P2RY12, TMEM119, AIF1, PTPRC* (CD45), *CD163* and *ITGA4* (CD49d) in glioma cells and non-malignant cells. Glioma single-cell RNA sequencing data were visualized using the UCSC Cell Browser (<https://cells.ucsc.edu>), under the Single-cell analysis of human glioma and immune cells identifies S100A4 as an immunotherapy target by (GSE182109) [65].


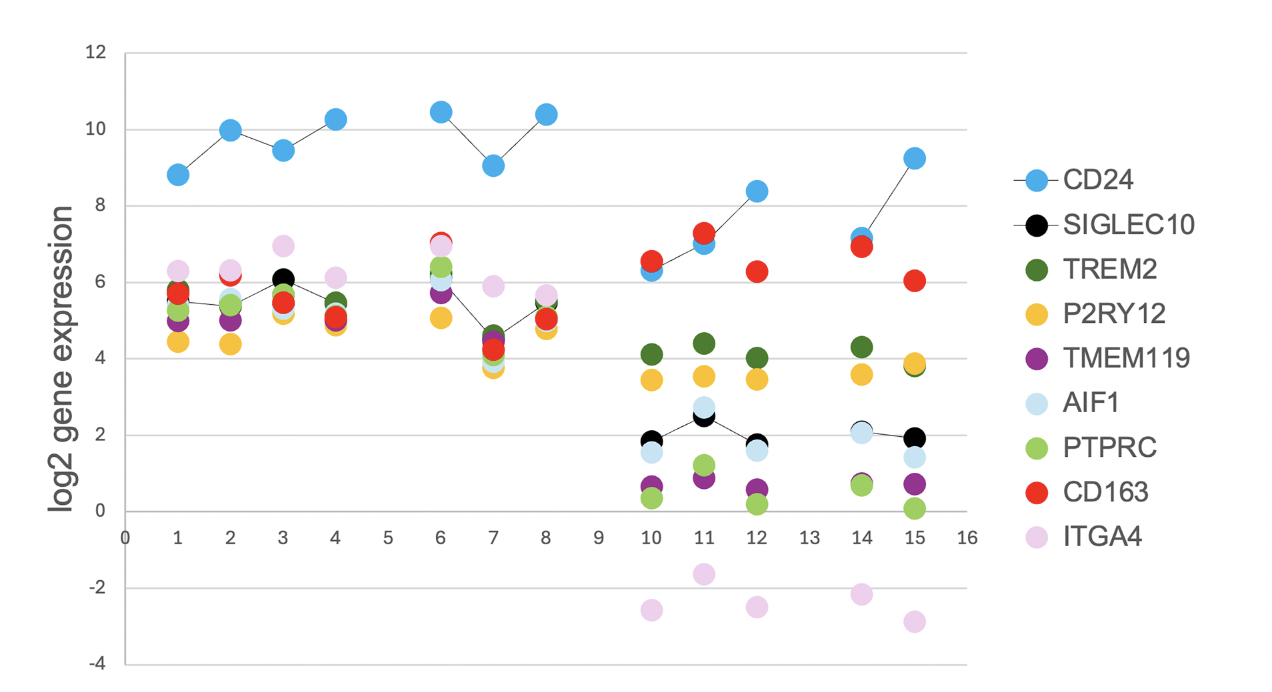


**Figure S2.** *CD24* gene expression in relation to TAM- and MG infiltration.

Dot plot showing mean log^2^ gene expression of selected markers *CD24, SIGLEC-10, TREM2, P2RY12, TMEM119, AIF1, PTPRC* (CD45), *CD163, ITGA4* (CD49d) across medulloblastoma (MB) and glioblastoma (GBM) subgroups. Each dot represents the mean expression for a marker within a subgroup. *CD24* and *SIGLEC-10* dots are connected by lines for emphasis. MB subgroups include WNT, SHH, Group 3, and Group 4 (Cavalli and Northcott datasets). GBM subgroups include GBM^C^, GBM^M^, GBM^PN^, IDHwt and IDHmut (TCGA dataset).

**
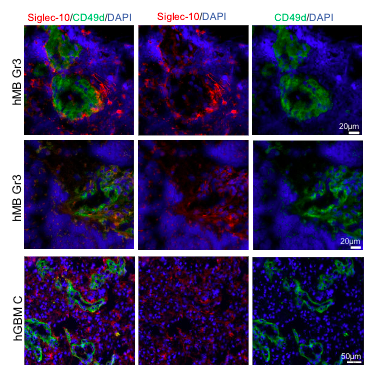
**

**Figure S3.** Immunohistochemical double-staining of tissue sections.

Double-labeling or single-labeling of Siglec-10 (red) and CD49d (ITGA4) (green) of frozen tumor sections (hMB; Gr3, hGBM; C). DAPI was used as nuclear staining. Representative pictures are shown. 20x (scale bar 50 µm) and 40x (scale bar 20 µm) magnification.


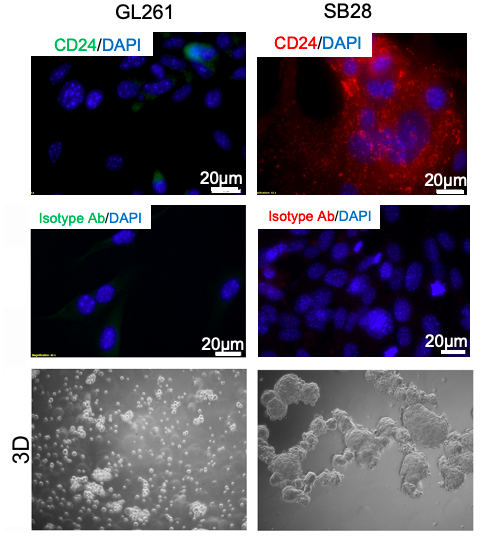


**Figure S4.** Adherent cells (2D) stained for CD24 or matched isotype antibody controls. GL261 (CD24green), SB28 (CD24red). DAPI was used as nuclear staining 40x magnification, Scale bar, 20µm. Cells cultured under stem cell conditions to form 3D-spheroids.


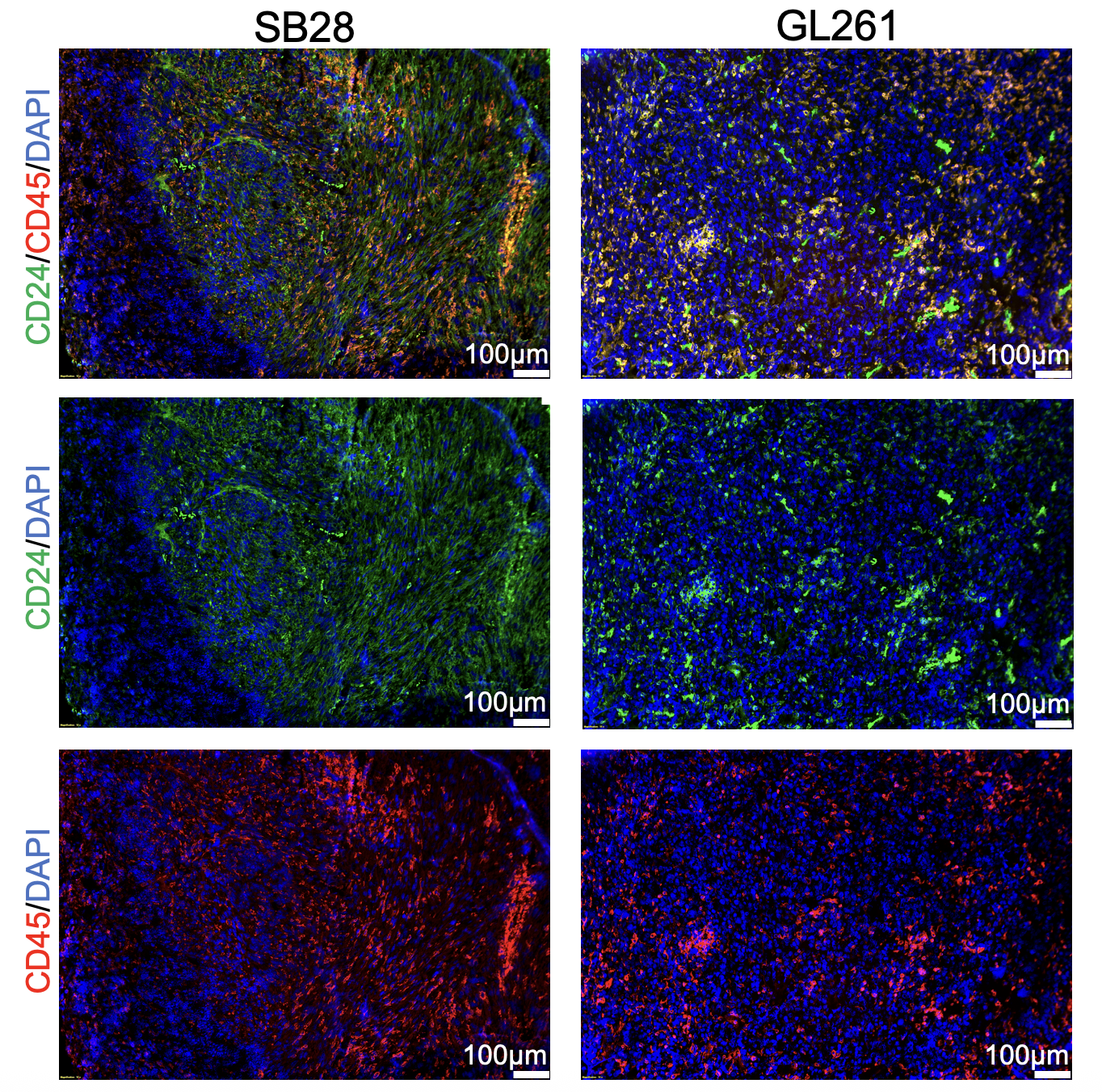


**Figure S5.** Immunohistochemical double**-**staining of CD24 and CD45 in tissue sections.

Upper panel, double labeling of CD24 (green) and CD45 (red) in SB28 and GL261 tumors. Double-labeled CD24^+^/CD45^+^ cells (yellow/orange). Middle panel, CD24 (green) only. Bottom panel, CD45 (red) only. DAPI was used as nuclear staining 10x magnification, Scale bar, 100µm.

**Material and methods**

**Single-cell RNA sequencing data**

MB single-cell RNA sequencing data was retrieved from the interactive Pediatric Neuro-oncology Cell Atlas browser (pneuroonccellatlas.org), under the Single-cell RNA-seq of childhood medulloblastoma samples (n=28) originally published by Riemondy et al., GEO dataset GSE156053 [64]. Glioma single-cell RNA sequencing data were visualized using the UCSC Cell Browser (<https://cells.ucsc.edu>), under the Single-cell analysis of human glioma and immune cells identifies S100A4 as an immunotherapy target by (GSE182109) [65].

**Immunohistochemistry of frozen tumor sections**

Brain tissue was frozen and fixed in cooled isopentane (-55ºC, VWR, Sweden) and kept thereafter at -80ºC until further analysis. Before staining, brain tissue was sectioned into 6µm-thick sections on a cryostat (CryoStar NX50, Epredia, Cellab, Sweden). Then, sections were fixed with acetone for 10 min. at room temperature, rehydrated with PBS without Ca^2+^ and Mg^2+^ (Gibco), blocked for 20min with 5% goat serum (Jackson ImmunoResearch, USA) and double stained for 60 min. Primary antibodies used were; rabbit-anti mouse/human Siglec-10 cat# BS-2707-R (ThermoScientific, Sweden), mouse anti-human CD49d(ITGA4) clone 9F10 cat#304302 (BioLegend), rat-anti mouse CD24 clone M1/69 cat#14-0242-85 (eBioscience™, Invitrogen, Sweden) and rat anti-mouse CD45-PE clone 30/F11 (BD Biosciences, BD Pharmingen™, Sweden). Sections were incubated for 30 min. with the following secondary antibodies; goat anti-rabbit Alexa 594, goat anti-mouse Alexa 488 and goat anti-rat Alexa 488 (MolecularProbes™, Invitrogen, Sweden). Matched isotype antibodies were used as controls. PBS were used to rinse the sections between the different steps followed by mounting in DAPI-containing mounting medium (ProLong™ Gold antifade, Invitrogen, Sweden). Pictures were taken at 20x and 40x magnification in the separate fluorescens-channels, and aligned pictures were created using the Cellsens Dimension software.

**Cell culture medium**

For 2D immunocytochemistry, mouse glioma cells (GL261, SB28) were cultured at 37 °C in the presence of 5% CO_2_ in R10-medium containing: RPMI 1640 medium supplemented with 2mM L-glutamine, 1 mM sodium pyruvate, 10mM HEPES, 50μg/mL gentamicin (GIBCO, fisher scientific, Sweden) and 10% fetal bovine serum (BIOWEST, VWR, Sweden). For 3D spheroid-formation, GL261 and SB28 cells were cultured in serum free cell culture medium (UltraCULTURE™, Lonza BioWhitaker Inc., VWR, Sweden supplemented with 2mM L-glutamine, 1% Penicillin-Streptomycin, Life Technologies, or with NeuroCult™ NS-A Proliferation kit (human), STEMCELL-technologies, Europe) supplemented with 50μg/mL gentamicin (GIBCO) with the addition of EGF 20ng/ml, and bFGF 20-40ng/ml (Chemicon, Merck Millipore, Sweden) in a 24-well plate without adherence (UltraLow™, Corning, Saveen & Werner AB, Sweden) and sphere-formation was observed.

**Immunocytochemical staining of CD24**

Immunocytochemistry was used to evaluate CD24 expression of GL261 and SB28 mouse glioma cells. Cells were cultured for 24 hours in 8-well chamber culture slides (BD Biosciences). Cells were fixed in 4% paraformaldehyde for 30 min, and blocked with 5% goat serum diluted in PBS for 20 min. Cells were incubated with the primary antibody (rat-anti mouse CD24 clone M1/69 cat#14-0242-85, LifeTechnologies, eBioscience™ for 2,5h at 37°C, followed by the secondary antibody, goat-anti rat Alexa 594 for 30 min at RT. The chamber slides were mounted wet using Pro-Long Gold anti-fading reagent with nuclear DAPI staining (Molecular Probes). PBS was used in all washing steps and as a diluent for reagents. Images were acquired using an Olympus BX-53 fluorescent microscope (LRI instrument AB) at 40X magnification.
